# B cell adapter for PI 3-kinase (BCAP) coordinates antigen internalization and trafficking through the B cell receptor

**DOI:** 10.1126/sciadv.adp1747

**Published:** 2024-11-15

**Authors:** Jonathan Lagos, Ursula Holder, Sara Sagadiev, Andrea Montiel-Armendariz, Lucy Z. Li, Chandrashekhar Pasare, Baidong Hou, Jessica A. Hamerman, Mridu Acharya

**Affiliations:** ^1^ Center for Immunity and Immunotherapies, Seattle Children’s Research Institute, Seattle, WA, USA.; ^2^ Center for Fundamental Immunology, Benaroya Research Institute, Seattle, WA, USA.; ^3^ Molecular and Cellular Biology Program, University of Washington, Seattle, WA, USA.; ^4^ Division of Immunobiology and Center for Inflammation and Tolerance, Cincinnati Children’s Hospital Medical Center, Cincinnati, OH, USA.; ^5^ Department of Pediatrics, University of Cincinnati, College of Medicine, Cincinnati, OH, USA.; ^6^ Institute of Biophysics, Chinese Academy of Sciences, Beijing, China.; ^7^ Department of Immunology, University of Washington, Seattle, WA, USA.; ^8^ Department of Pediatrics, University of Washington, Seattle, WA, USA.

## Abstract

B cell adapter for PI 3-kinase (BCAP) is an adaptor molecule associated with signaling through multiple immune receptors, including the B cell receptor (BCR). However, B cell–intrinsic role of BCAP in antibody responses is unclear. We investigated the role of BCAP in B cell response to viral particles and found a previously unidentified mechanism by which BCAP regulates antigen-specific responses. B cell–specific deletion of BCAP in mice leads to decreases in antigen-specific responses through defects in BCR-antigen endocytosis. BCAP is necessary to orchestrate actin reorganization around the antigen for efficient endocytosis through BCR and intracellular processing of antigens. Therefore, loss of BCAP from B cells leads to defects in antigen endocytosis, hampering the propagation of antigen-derived signals and decreasing the ability of B cells to present antigens to T cells. Thus, our study clarifies how BCAP regulates B cell responses to complex antigens and elucidates that antigen positioning inside B cells determines different B cell activation outcomes.

## Teaser

BCAP is necessary to internalize BCR-bound antigens and antigen trafficking, BCR signal propagation, and antigen presentation.

## INTRODUCTION

An early step in establishing B cell immune responses is antigen engagement by the B cell receptor (BCR) on naïve cells, triggering transmembrane signaling and antigen internalization. This event can be modulated by additional receptors, such as Toll-like receptors (TLRs) activated by innate immune signals associated with the antigen, as well as co-receptors and adaptor proteins, which are involved in fine-tuning signal propagation through the BCR. Integration of these signals from multiple receptors and adaptor proteins is key for adequate signal transduction and appropriate B cell response to antigens. Signaling through these receptors has been investigated in B cells, but it is still unclear how signals initiated through these receptors are organized inside the cells for appropriate immune response.

B cell adapter for PI 3-kinase (BCAP) is a protein first identified as an adaptor molecule that binds to the p85 subunit of phosphoinositide 3-kinase (PI3K) (*1*). BCAP is tyrosine phosphorylated by Syk and Btk, which provides binding site(s) for the p85 subunit of PI3K. Initial studies showed that BCAP-deficient mice have defects in B cell development and activation. These mice exhibit a slightly reduced number of follicular B cells, a reduction in B1 B cells, and a defect in mounting T-independent B cell responses after immunization (*2*). BCAP-deficient B cells also show impairment in BCR-induced Ca^2+^ flux, proliferation, and nuclear factor κB (NF-κB) activation (*3*), indicating that BCAP is involved in BCR signaling in B cells. While studies on a chicken B cell line had shown that BCAP is involved in BCR-induced PI3K activation, mouse studies showed that the defects in BCR-induced Ca^2+^ flux in the BCAP^−/−^ cells were not attributable to an impairment of BCR-mediated PI3K activation, as BCAP deletion in B cells did not lead to defects in PI3K activation (*2*). Moreover, a later study showed that deletion of both BCAP and CD19 in B cells leads to a block in BCR-mediated Akt activation, suggesting that other molecules, such as CD19, could rescue the effects of BCAP deletion on PI3K activation (*4*). Thus, it is still unclear how BCAP is mediating its effects on BCR signaling and BCR-mediated B cell proliferation. Similarly, immunization studies in BCAP knockout (KO) mice have not elucidated a B cell–specific role of BCAP or the role of BCAP in the humoral response to more complex antigens, such as viral particles, which can engage TLRs in addition to the BCR.

BCAP has also been shown to play essential roles in other immune cell subsets. We and others have shown that BCAP is required for TLR-mediated PI3K activation in macrophages (*5*, *6*), and, in plasmacytoid dendritic cells (pDCs), BCAP promotes TLR-induced interferon-α production via effects on PI3K, Rac1, and inhibitor of nuclear factor κB kinase α (IKKα) activation (*7*). BCAP also has cell-intrinsic roles in T cells; BCAP is involved in CD8^+^ T cell activation and effector/memory differentiation (*8*) and promotes CD4^+^ T helper 17 differentiation (*9*). Both PI3K and TLR signaling are important features of B cell activation. Furthermore, PI3K/Rac1 axis–mediated actin cytoskeleton remodeling has been implicated in the internalization of large particles in B cells (*10*). On the basis of these roles of BCAP on BCR and TLR signaling and actin remodeling, we asked whether BCAP has a role in regulating B cell responses to more complex antigens such as viral particles. Viral particles can ligate both BCR and TLRs and require appropriate intracellular trafficking events to initiate optimal signals through these receptors. Therefore, these particles allowed us to probe the role of BCAP in regulating both signaling and intracellular trafficking events.

Our data indicate that BCAP orchestrates the organization of BCR-associated antigen signals and subsequent antigen processing through actin remodeling. This role of BCAP during early B cell activation by antigen has lasting consequences for the expansion of antigen-specific B cells and humoral response as well as in the presentation of antigen by B cells to T cells.

## RESULTS

### BCAP has a B cell–intrinsic role in regulating humoral response against viral antigens

**Fig. 1.**
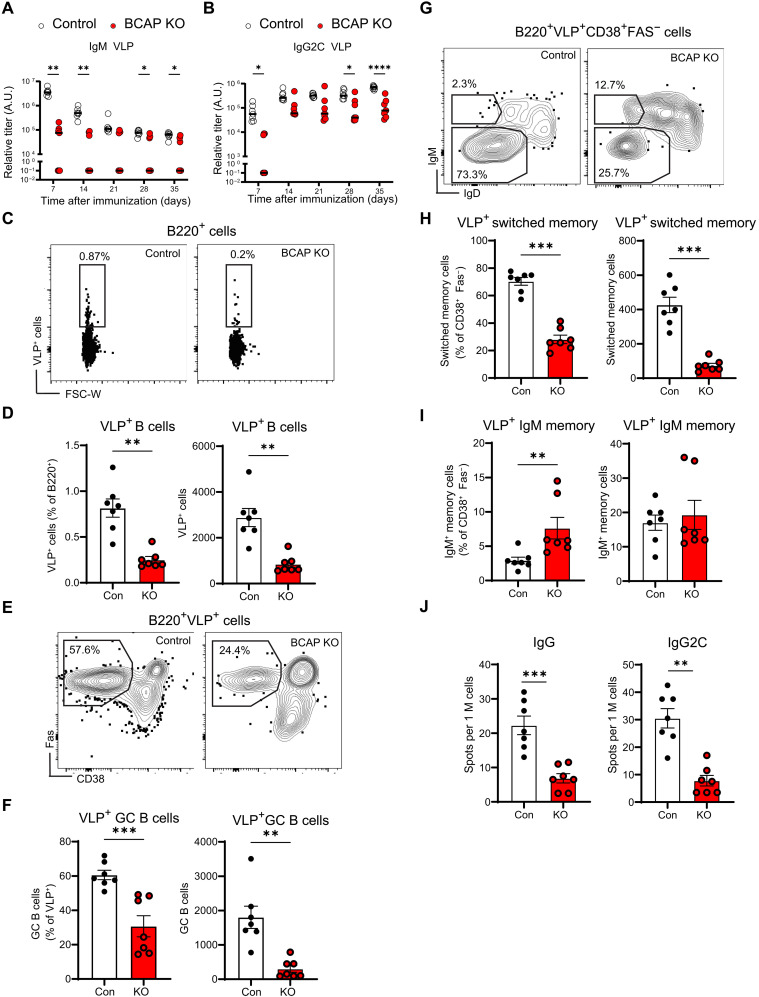
BCAP KO B cells show decreased antigen-specific B cell response. (**A** and **B**) Serum anti-VLP antibody titers in control and BCAP KO mice immunized with 2 μg of VLPs containing ssRNA measured at 7, 14, 21, 28, and 35 days after immunization. (**C**) Fluorescence-activated cell sorting (FACS) plots of VLP-immunized control and BCAP KO mice splenocytes stained with fluorescent VLPs, showing VLP^+^ cells in the B220^+^ gate. FSC-W, forward scatter width. (**D**) Proportion and count of spleen VLP^+^ B cells [gated as in (C)] from control and KO mice immunized with 2 μg of VLPs for 35 days. (**E**) B220^+^VLP^+^ GC B cells were identified as Fas^+^CD38^−^ cells in control and KO mice. (**F**) Proportion and count of spleen VLP-specific GC B cells [gated as in (E)] from control and KO mice. (**G** to **I**) Splenocytes were gated as B220^+^VLP^+^Fas^−^CD38^+^ non-GC B cells, and the frequencies and count of switched memory and IgM memory B cells were determined on the basis of IgM and IgD levels. (**J**) VLP-specific plasma cells of IgG or IgG2c isotype enumerated by ELISpot assay on bone marrow (BM) cells from control or KO mice harvested at day 35 after immunization. All data are from one of the two independent experiments yielding similar conclusions. Points represent individual mice (*n* = 7 mice per group) with mean [(A) and (B)] or mean with SEM [(D) to (J)] shown. *P* values of less than 0.05 are shown: *P < 0.05, **P < 0.01, ***P < 0.001, and ****P < 0.0001, calculated with two-way analysis of variance (ANOVA) with multiple comparisons test [(A) and (B)] or Mann-Whitney *U* test [(D) to (J)]. A.U., arbitrary units.

Previous studies have shown a role for BCAP in regulating T-independent antibody responses (*2*), but mechanisms for this and the precise role of BCAP in antibody responses are not clear. To further clarify the role of BCAP in humoral responses, we immunized control and BCAP KO mice with diverse antigens. We first evaluated the humoral response to T cell–dependent antigen nitro-phenol-haptenated chicken γ-globulin (NP-CGG) with alum as adjuvant and found slightly lower titers of NP-specific immunoglobulin M (IgM) and IgG antibodies at days 14 and 21 in BCAP KO mice compared to those in control mice (fig. S1, A and B). This difference in antibody titers was attenuated at later time points, suggesting a transient defect in the early humoral response. To understand the role of BCAP in antibody responses to more complex antigens, we immunized mice with virus-like particles (VLPs) derived from bacteriophage Qβ capsid proteins (*11*). Qβ-VLP incorporates single-stranded RNA (ssRNA) within the viral capsids and induces strong germinal center (GC)–derived antibody responses, dependent on B cell–intrinsic MyD88 signaling (*12*–*14*). In response to VLP immunization, BCAP KO mice showed decreases in both early and late IgM antibody titers compared to control mice (Fig. 1A). The TLR-dependent IgG2c antibody titers were also significantly decreased in the BCAP KO mice at multiple time points (Fig. 1B). We also analyzed the expansion of VLP-specific B cells at day 35 after immunization, making use of fluorescent Qβ-VLP which can be used to identify VLP specific cells induced by immunization (*13*). BCAP KO mice showed fewer VLP-specific B cells compared to controls (Fig. 1, C and D) and a lower proportion of GC B cells (Fas^+^CD38^−^) within the VLP-specific B cell compartment (Fig. 1, E and F). We also found fewer VLP-specific switched memory B cells (Fig. 1, G and H). These differences in the B cell population were seen with both percentages of cells within specific compartments and when comparing cell counts in each compartment. In contrast, there was an increase in the percentage, but not in the number, of IgM memory B cells in the BCAP KO mice (Fig 1I). Moreover, the effect of loss of BCAP also extended to long-lived plasma cells, as we found fewer VLP-specific IgG- and IgG2c-secreting plasma cells in the BCAP KO bone marrow (BM), at day 35 after immunization (Fig. 1J). To confirm this decreased response to TLR ligand associated viral antigens in the BCAP KO mice, we immunized mice with inactivated H1N1 Puerto Rico 8 (iPR8) virus and observed a similar trend, with a reduction in virus-specific IgM, IgG, and IgG2c antibody titers at 28 to 35 days in the BCAP KO mice (fig. S1, C and D). The coadministration of antigen and TLR ligand through use of a vaccine-grade formulation of the TLR7 agonist imiquimod (imiquimod-SE) mixed with NP-CGG did not show a reduction in the titer of antigen-specific IgM or IgG2c antibodies between control and BCAP KO mice (fig. S1, F and G), suggesting that the differences seen with VLP and iPR8 immunization in the BCAP KO mice are driven by changes in the capacity of the B cell to process TLR-associated antigens rather than defects in TLR recognition. Thus, loss of BCAP leads to defects in B cell responses to TLR ligand associated complex antigens via defects in the generation of antigen-specific GC, memory B cells, and long-lived plasma cells.

**Fig. 2.**
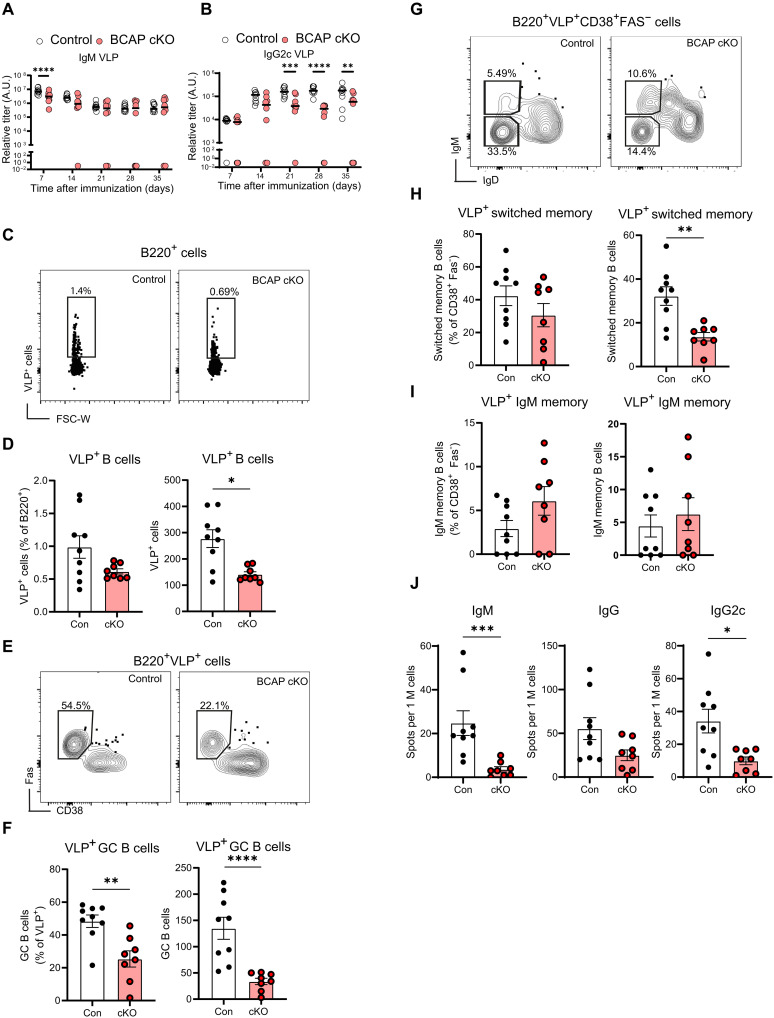
Decreased humoral antigen-specific response in BCAP KO mice is due to B cell–intrinsic defect. (**A** and **B**) Serum anti-VLP antibody titers in control and BCAP cKO mice immunized with 2 μg of VLPs containing ssRNA measured at 7, 14, 21, 28, and 35 days after immunization. (**C**) FACS plots from splenocytes of VLP-immunized control and cKO mice stained with fluorescent VLPs showing VLP^+^ cells in B220^+^ gate. (**D**) Proportion and count of spleen VLP^+^ B cells [gated as in (C)] from control and BCAP cKO mice immunized with 2 μg of VLP for 35 days. (**E**) B220^+^VLP^+^ GC B cells were identified as Fas^+^CD38^−^ cells in control and BCAP cKO mice. (**F**) Proportion and count of spleen VLP-specific GC B cells [gated as in (E)] from control and BCAP cKO mice. (**G** to **I**) Splenocytes were gated as B220^+^VLP^+^Fas^−^CD38^+^ non-GC B cells, and the frequencies and count of switched memory and IgM memory B cells were determined on the basis of IgM and IgD levels. (**J**) Antigen-specific plasma cells enumerated by ELISpot assay on BM cells from control or BCAP cKO mice harvested after immunization. Data from two independent experiments were combined, representative of three different experiments. All data points represent individual mice (*n* = 9 for control and *n* = 8 for cKO mice per group) with mean [(A) and (B)] or mean with SEM [(D) to (J)] shown. *P* values of less than 0.05 are shown: *P < 0.01, **P < 0.001, ***P < 0.001, and ****P < 0.0001, calculated with two-way ANOVA with multiple comparisons test [(A) and (B)] or Mann-Whitney *U* test [(D) to (J)].

Previous studies have shown that BCAP plays an important role in multiple immune cell types, such as macrophages (*5*, *6*), conventional dendritic cells (cDC) (*15*), pDC (*7*), CD4, and CD8 T cells (*8*, *9*). Therefore, to determine whether the decreased responses to viral antigens seen in the BCAP KO mice are due to the effects of loss of BCAP from B cells, we generated mice with BCAP deletion only in B cells, by crossing *Cd19^cre^* mice to *Pik3ap1^fl/fl^* (BCAPfloxed) mice (*9*). We assessed B cell–specific deletion of BCAP in these conditional KO mice by flow cytometry and found variability in BCAP deletion. Therefore, we screened for BCAP deletion in peripheral blood B cells by flow cytometry and used the conditional BCAP KO mice with 80% deletion of BCAP in B cells, for in vivo studies (fig. S2A). We did not find major changes in the number or percentages of total B cells in mice with B cell–specific BCAP deletion compared to those in control mice, although there was a small decrease in the percentage of follicular B cells and an increase in marginal zone B cells (fig. S2, B to E). We also did not find differences between control and KO mice in TLR7 or TLR9 levels, as measured by quantitative polymerase chain reaction (qPCR) from follicular B cells (fig. S2F). Conditional BCAP KO (cKO; *Cd19^cre/+^Pik3ap1^fl/fl^*) mice and matched control mice (*Cd19^cre/+^Pik3ap1^+/+^*) were immunized with VLP, and B cell responses were analyzed as in Fig. 1. BCAP cKO mice exhibited a reduction in IgM antibody titers at 7 days and a decrease in IgG2c antibody titers at multiple time points after immunization compared to control mice (Fig. 2, A and B). Flow cytometry analysis for antigen-specific cells at day 35 showed a decrease in VLP-specific B cells and a significant decrease in the frequency and number of VLP-specific GC B cells in the cKO mice compared to those in controls (Fig. 2, C to F). We also found a decrease in antigen-specific switched memory B cells (Fig. 2, G and H) and a trend toward an increase in IgM memory B cells in the BCAP cKO mice (Fig. 2, G and I). VLP-specific plasma cells of the IgG, IgG2c, and IgM isotype were also decreased in BCAP cKO BM compared to those in the controls (Fig. 2J). These changes were not attributable to differences in CD19 because control and cKO mice present similar levels of CD19 after immunization (fig. S2G). We also used the variability in BCAP levels to confirm the role of BCAP in the generation of antigen-specific B cells. We immunized mice with BCAP deletion in 70% of B cells with VLP and found that ~80% of VLP-specific B cells and GC cells were B cells that maintained BCAP expression, indicating a competitive advantage for the BCAP-expressing cells to become activated through their BCR and enter the GC compared to that for the BCAP-deleted B cells (fig. S2H). In addition, we immunized control and BCAP cKO mice with NP-CGG with alum adjuvant to compare the response to this antigen with the defects in the VLP response. We found a nonsignificant slight reduction in NP-specific IgG1 antibody levels at early time points in the cKO mice (fig. S3A). We also observed a nonsignificant decrease in the antigen-specific IgM and IgG2c plasma cells in the BM as measured by enzyme-linked immunosorbent spot (ELISpot) in the cKO mice (fig. S3, B and C). These findings are all similar to the findings from the global BCAP KO mice (Fig. 1 and fig. S1, A and B) and thus confirm that BCAP has a B cell–intrinsic role in regulating humoral responses, which is especially relevant for response to complex virus–derived antigens.

### BCAP regulates B cell proliferation in response to BCR stimulation

Next, we sought to understand the mechanisms by which BCAP regulates B cell responses to complex antigens. The decrease in antibody response and antigen-specific cells seen in BCAP KO mice could be due to defects in differentiation or due to defects in the proliferation/expansion of B cells. We first investigated whether BCAP affects the capacity of B cells to differentiate, using an in vitro assay described previously (*16*, *17*). This assay leads to the differentiation of B cells into antibody-secreting cells (ASCs) and also allowed us to investigate differentiation into activated B cell stages.

**Fig. 3.**
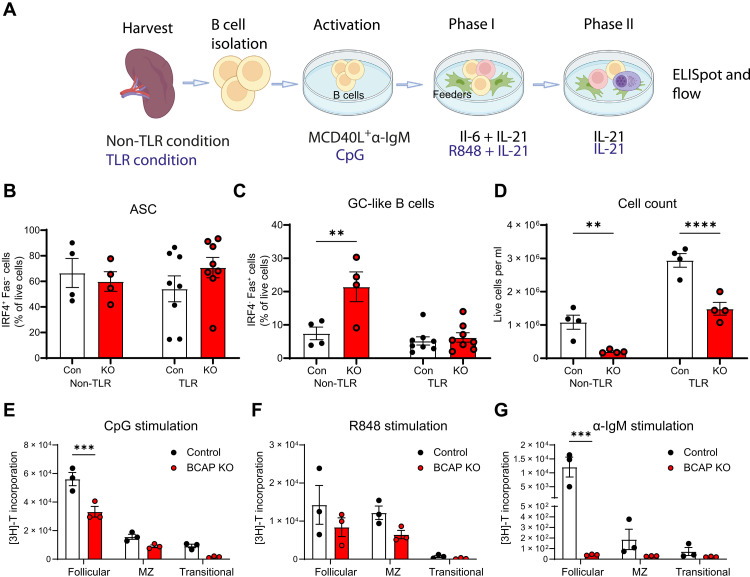
BCAP KO B cells show defects in proliferation in response to BCR activation. (**A**) Experimental workflow of the plasma cell differentiation assay. This workflow includes a three-step differentiation culture that is divided by a B cell activation phase with or without the presence of TLR ligands, a plasmablast differentiation phase (phase I), and a plasma cell differentiation phase (phase II) using the different cocktails of soluble factors and cytokines specified. (**B**) Bar graph showing the percentage of IRF4^+^FAS^−^ antibody-secreting cells (ASCs) from B220^+^ gate 8 days after activation. Every point is a biological replicate expressed as means ± SEM (*n* = 4 in non-TLR conditions and *n* = 8 in TLR conditions). (**C**) Bar graph showing the percentage of Fas^+^IRF4^−^ GC-like B cells from B220^+^ gate 8 days after activation. Every point is a biological replicate expressed as means ± SEM (*n* = 4 in non-TLR conditions and *n* = 8 in TLR conditions). (**D**) Cell count and viability assay were used on the last day of differentiation culture. Every point is a biological replicate expressed as means ± SEM (*n* = 4). (**E** to **G**) Sorted spleen follicular (B220^+^CD24^+^CD23^hi^CD21^+^), marginal zone (MZ; B220^+^CD24^+^CD23^low^CD21^hi^), and transitional B cells (B220^+^CD21^low^CD24^hi^) from BCAP-KO and control mice after TLR ligands (CpG-C, 2 μM; and R848, 5 μg/ml) or α-IgM (10 μg/ml) stimulation. Proliferation was measured by [3H]-thymidine incorporation and is expressed as means ± SEM for three independent cultures. *P* values of less than 0.05 are shown: *P < 0.05, **P < 0.01, ***P < 0.001, and ****P < 0.0001 by two-way ANOVA with multiple comparisons test. (A) created using BioRender.com.

B cells isolated from mouse spleen were activated either with MegaCD40 ligand (MCD40L) and anti-IgM (α-IgM), as non-TLR condition that includes BCR stimulation, or with CpG, as TLR stimulation condition. Cells were then cultured with different cytokines and/or TLR ligands to allow B cell differentiation (Fig. 3A). Both TLR and non-TLR stimulation conditions were used to understand whether the loss of BCAP affects B cell function through either the BCR or TLR-mediated pathways or both. On day 8 after culture, we analyzed differentiated cells by flow cytometry (fig. S4A) and ELISpot assay. We did not observe a significant difference in IgM, IgG, or IgG2c ASCs generated from control or BCAP KO B cell cultures, as measured by ELISpot (fig. S4, B to D) or when we identified ASCs by flow cytometry as IRF4^+^Fas^−^ cells (Fig. 3B). In addition, differentiation of B cells into more activated GC-like cells indicated by Fas expression was also not defective in the BCAP KO cultures. We observed an increase in percentages of these cells from the BCAP KO cultures compared to control cultures with BCR stimulation (Fig. 3C). However, we found that BCAP KO cultures consistently showed decreased number of live cells at the end of the cultures, suggesting a defect in the expansion or proliferation of the BCAP KO cells (Fig. 3D). To further understand this defect in BCAP KO B cells, we measured B cell proliferation after BCR or TLR stimulation through a thymidine incorporation assay. B cells from the spleen were sorted by flow cytometry as follicular, transitional, or marginal zone B cells based on surface marker expression and stimulated with TLR ligand, BCR ligand, or a combination of both. We assessed B cell subsets separately to confirm that the defects observed were due to stimulation and not due to changes in proportions of B cell subpopulations in BCAP KO spleens. All three subpopulations of BCAP KO B cells showed a decrease in proliferation in response to CpG (Fig. 3E), and a trend toward decreased proliferation was also seen with R848 (Fig. 3F). However, the most notable decrease in proliferation was seen in the follicular B cell subset after IgM stimulation with almost no proliferation seen in the BCAP KO cells in this setting (Fig. 3G). This decrease in response to IgM simulation was still observed when cells were stimulated with both BCR and TLR ligands, but this was less marked than the difference observed with BCR stimulation alone (fig. S4, E and F). Together, these data show that BCAP KO B cells do not have defects in differentiating into activated B cells, ASC, or in secreting antibodies. However, they have a defect in their ability to expand in response to TLR or BCR stimulation, particularly their ability to expand in response to BCR activation is severely compromised.

### Loss of BCAP leads to altered BCR-induced signal localization

**Fig. 4.**
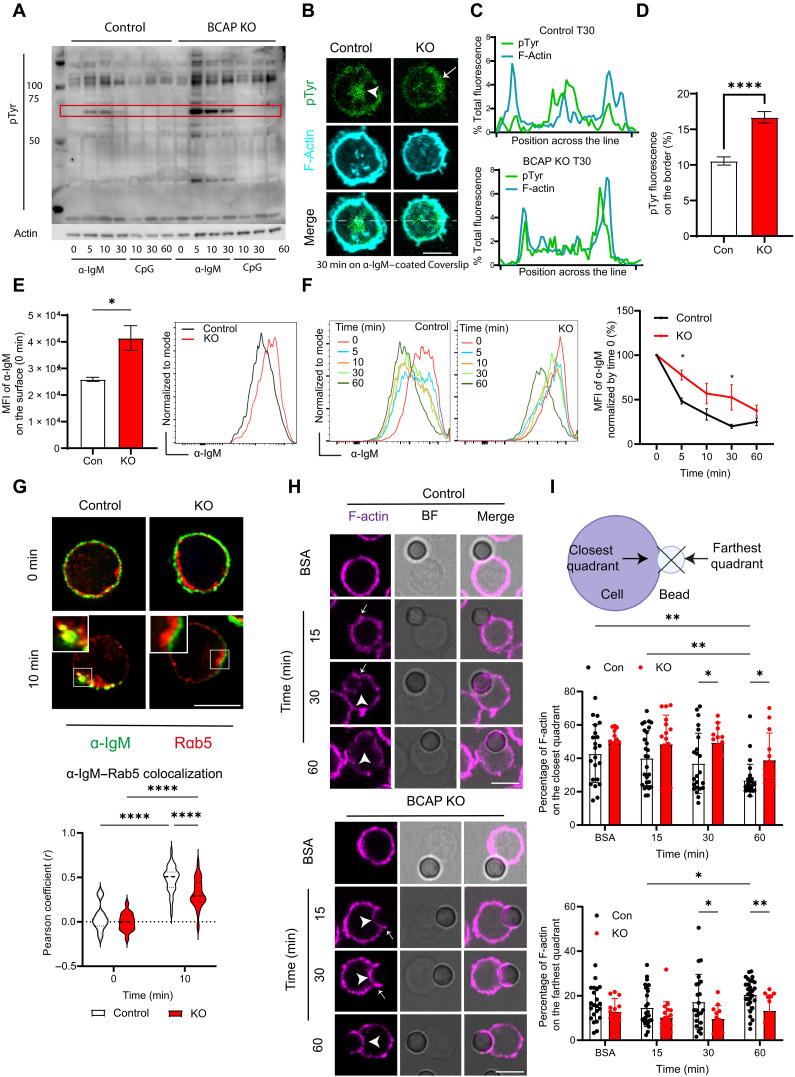
BCAP KO B cells exhibit increased global BCR signaling but decreased BCR endocytosis. (**A**) Immunoblotting of pTyr from control or BCAP KO splenic B cells lysates stimulated with α-IgM or CpG for the indicated time points. Bands within the red box are quantified in fig. S5A. *n* = 3. (**B**) Representative confocal planes showing the immune synapse of B cells activated on α-IgM–coated coverslips stained for pTyr (green) and F-actin (cyan). Arrowhead, central pTyr; arrows, peripheral pTyr. (**C**) Normalized fluorescence intensity distributions of pTyr (green) and F-actin (cyan) sampled across the outlined portion of the images in (B). (**D**) Graph represents pTyr fluorescence percentage in the peripheral ^1^/_4_ of the cell defined by actin. Data of two independent experiments yielding similar findings (221 to 214 cells per condition). (**E**) Histograms and graph surface α-IgM of splenic B cells from control and KO mice at resting condition. *n* = 4, means ± SEM. (**F**) Histograms and line graph of α-IgM remaining on surface (percentage from α-IgM MFI at time 0). *n* = 3, means ± SEM. (**G**) Confocal single plane of BCAP KO or control B cell incubated with α-IgM–biotin (green) and stained with Rab5 (red). Scale bar, 5 μm. Graph showing Pearson coefficient between α-IgM and Rab5 analyzed in multiple *z*-stack using ImageJ. Data are shown as means ± SEM of one of the two independent experiments, yielding similar results (52 to 59 cells per condition). (**H**) Representative confocal plane of control or KO splenocytes incubated with BSA or α-IgM–coated beads for different time points. Cells were fixed and stained for F-actin (magenta). Arrowheads, base of actin cup. BF, bright field. (**I**) Quantification of (H). Data from two experiments combined (91 to 55 cells per condition). *P < 0.05, **P < 0.01, and ****P < 0.0001 were calculated using Mann-Whitney *U* test [(D) and (E)] or two-way ANOVA with multiple comparisons test [(F), (G), and (I)].Schematic in (I) created using BioRender.com.

Our data indicated that BCAP KO B cells cannot respond effectively to BCR stimulation. This is similar to previous studies that have shown defects in BCR-induced Ca^+2^ flux and response to IgM stimulation in the BCAP KO B cells (*2*). However, in these previous studies, PI3K activity was shown to be conserved in the BCAP KO B cells, so the mechanisms by which BCAP regulates responses to BCR stimulation remain unclear. Therefore, we next addressed why BCAP KO B cells might be less responsive to BCR stimulation. First, we evaluated overall signaling in BCAP KO B cells after BCR stimulation by measuring protein phosphorylation via assessment of phospho-tyrosine (pTyr) by Western blot. In control cells, we observed tyrosine phosphorylation of multiple proteins after α-IgM stimulation of the BCR. To our surprise, we found increased intensity of pTyr signal, in multiple proteins in the BCAP KO cells, indicating an increase in overall tyrosine phosphorylation of proteins in the BCAP KO B cells after BCR stimulation (Fig. 4A). This increase in phosphorylation of proteins in the BCAP KO cells was more obvious with IgM stimulation than with CpG stimulation (Fig. 4A and fig. S5A), indicating preferential effects of BCAP deletion on BCR signaling. Because of this finding and the profound effects of BCAP deficiency on BCR-induced B cell proliferation (Fig. 3G), we focused our subsequent studies on understanding how BCAP regulates responses to BCR stimulation.

The discrepancy between the increase in overall BCR-induced pTyr and the decrease in BCR-induced proliferation in the BCAP KO B cells indicated that there could be issues with the organization or localization of BCR signals in the absence of BCAP. Therefore, we decided to use microscopy for a detailed analysis of intracellular localization of BCR-induced signals in control and BCAP-deficient B cells. To investigate the overall distribution of signals inside the cells after BCR stimulation, we used cells plated on coverslips coated with α-IgM antibody to simulate antigen stimulation of the BCR and followed the pTyr signal by confocal microscopy. This system allowed us to follow the distribution of signal in an immune synapse-like platform delimited by F-actin. In control cells, we expected a higher concentration of the antigen-induced pTyr signal in the central area of the cell, where the cell surface contacts with antigen, as this is the area that likely corresponds to the central supramolecular activation complex (*18*), and this is what we observed at 30 min after plating the cells on α-IgM, when the cell is fully spread (Fig. 4B). However, we observed a different pattern of localization of pTyr signal in the BCAP KO cells, which showed more pTyr signals on the border of the cell in F-actin–rich area and very little signal in the central areas (Fig. 4, B to D). Thus, despite a quantitative increase in BCR stimulation–induced pTyr signal in BCAP KO B cells, the intracellular distribution of this signal is altered in these cells with localization of signals in peripheral areas of the cells.

### BCAP orchestrates actin reorganization required for efficient BCR endocytosis

Because defects in BCR stimulation–induced signal distribution could be related to antigen and BCR endocytosis (*19*), we investigated the ability of BCAP KO B cells to internalize the BCR after soluble α-IgM stimulation, using biotinylated α-IgM which can be detected by fluorescent streptavidin and flow cytometry. We first evaluated surface BCR levels using α-IgM to stain for the BCR in control and BCAP KO cells at 4°C and found higher BCR levels on the BCAP KO cell surface in resting conditions (Fig. 4E). To follow BCR endocytosis, B cells were stimulated with biotinylated α-IgM and allowed to internalize the α-IgM–BCR complex at 37°C. At different time points, cells were then stained with fluorescent streptavidin, which labels the α-IgM–stained BCR remaining on the surface. Thus, decreases in fluorescence of surface α-IgM at different time points was used to evaluate BCR internalization. At 5 min after stimulation, we detected that ~60% of the BCR was internalized in control B cells, but only ~30% of the BCR was internalized in KO cells. At later time points, in the control B cells, almost ~80% of the BCR was internalized, whereas, in BCAP KO cells, only ~50% of the BCR was endocytosed (Fig. 4F).

We further confirmed this reduced BCR endocytosis in BCAP KO cells by microscopy using a similar approach of stimulation of cells with soluble biotinylated α-IgM. We measured the localization of the surrogate antigen, α-IgM, in early endosomal compartments by staining with the early endosomal marker Rab5. We observed a significant increase in α-IgM that was internalized and localized into early endosomal Rab5^+^ compartments in control cells at 10 min after stimulation. In BCAP KO B cells, there was some increase in colocalization between α-IgM and Rab5, but this increase was lesser than what was seen in control cells, indicating defects in antigen and BCR trafficking to early endosomal compartments in the KO B cells (Fig. 4G). We further evaluated the kinetics of antigen and BCR localization at different time points by quantifying α-IgM fluorescence in concentric circles from the center to the periphery of the cell and plotted this as a function of the distance of α-IgM fluorescence from the cell center (fig. S5B). In control B cells, the fluorescence of α-IgM changes its distribution, starting from the periphery to accumulating in the center of the cell at later time points. However, in BCAP KO B cells, fluorescence continues to build up near the cell boundaries over the different time points (fig. S5, C and D). To elucidate these changes in α-IgM internalization with better resolution, we performed expansion microscopy with control and BCAP KO B cells at 10 min after fluorescent α-IgM stimulation. We also included staining of the plasma membrane using the wheat germ agglutinin (WGA) marker to further clarify antigen internalization. The increased resolution provided by expansion microscopy showed that, in control cells, α-IgM fluorescence intensity is observed inside the cell between the nucleus and the plasma membrane at 10 min after stimulation (fig. S5E, arrowhead). In contrast, in the BCAP KO cells, almost all α-IgM was concentrated in the border of the cells together with the WGA marker (fig. S5E, arrow), confirming reduced BCR-mediated antigen endocytosis in the BCAP KO B cells. Furthermore, we confirmed that this BCR and antigen internalization is dependent on PI3K activity as we also observed decreased BCR internalization in control cells treated with the PI3K inhibitor ZSTK474 measured as the change in percentage of α-IgM^+^ cells (fig. S5, F and G). To investigate whether this reduced BCR-mediated endocytosis in BCAP KO B cells is also relevant for a bigger and more complex antigens, we used a fluorescent streptavidin bead conjugated with α-IgM and measured the capacity of B cells to internalize these beads by microscopy. We compared the internalization of soluble antigens with internalization antigen-conjugated beads by measuring the fluorescence of the antigen inside and outside the cell. We defined an area of up to 120% of the cell radius and divided outside and inside of the cell by the cortical actin (for beads) or the bright-field contour (for soluble antigen). We then measured the fluorescence of the antigen using the radial scan plugin in ImageJ. With soluble antigen at 10 min after stimulation, control cells showed ~80% of the antigen inside the cell, while, in BCAP KO cells, soluble antigen internalization was reduced with ~70% of the antigen inside the cell. With bead-conjugated antigen, control cells had internalized ~50% of the antigen, while BCAP KO cells had internalized only ~20% (fig S5, H and I). These data establish that defects in antigen endocytosis in the BCAP KO B cells are relevant for both soluble and larger, more complex antigens. However, these defects are aggravated with more complex antigens.

We next focused on the mechanism leading to impaired antigen-BCR endocytosis in BCAP KO cells. Actin dynamics is known to be crucial for BCR endocytosis (*20*), and BCAP has previously been linked with actin polymerization (*7*, *21*). Therefore, we asked whether alterations in actin reorganization around the antigen are related to changes in endocytosis in the BCAP KO B cells. To investigate actin reorganization around an antigen, we incubated B cells with α-IgM–coated beads that allow us to focus on a discreet point of contact between the cell and the antigen. In control cells, actin assembly begins with a marked accumulation of F-actin around developing cups that mark the initial contact area between the cell and the bead. This is followed by the extension of the actin cytoskeleton along the perimeter of the bead and the clearance of F-actin from the base of the cup (Fig. 4H, arrowhead). The early phase of actin assembly at the developing cup was also observed in BCAP KO cells, but clearance of F-actin was not observed in the base of the cup, where polymerized actin persisted for extended periods (Fig. 4H, arrow). We quantified F-actin around the bead through a quadrant picking plugin in ImageJ, which divides the area of the bead into four quadrants. We measured the fluorescence of F-actin in these four quadrants and calculated the percentage of F-actin remaining in those quadrants through time (Fig. 4I). In control cells, we observed F-actin concentrated at the closest quadrant to the cell at initial time points, decreasing at later time points. Meanwhile, F-actin increased at the quadrant farthest from the cell. In comparison, BCAP KO B cells showed an increased percentage of F-actin in the closest quadrant, and it remained there through time (Fig. 4I). These data indicate that BCAP is dispensable for actin assembly around the antigen but necessary for the secondary actin clearance accompanying BCR antigen internalization. We also evaluated whether actin polymerization and clearance are affected in BCAP KO B cells in response to endocytosis of soluble antigens. At 60 min of internalization, we measured F-actin within a circle of a 1.5 μm radius, with the antigen as a center. We measured the relationship of F-actin fluorescence with distance from the antigen and found that, in control cells, F-actin is concentrated in the pixels farthest from the antigen, while, in BCAP KO cells, F-actin is concentrated closest to the antigen (fig. S6, A and B). Thus, BCAP coordinates actin reorganization in areas of BCR antigen encounter for efficient internalization and intracellular positioning of BCR signals.

### BCAP orchestrates actin organization through recruitment of actin-regulatory proteins

**Fig. 5.**
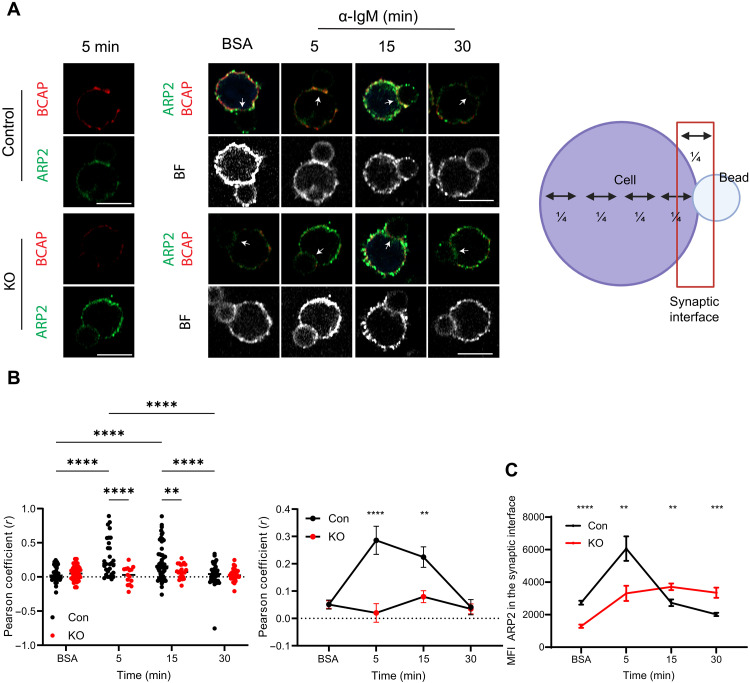
BCAP recruits actin-regulatory proteins to the antigen. (**A**) Confocal images showing Arp2 and BCAP localization in cells stimulated with an antigen-coated bead (α-IgM) or a negative ligand (BSA). Left images show one plane of control and BCAP KO B cell for BCAP (red) and Arp2 (green) staining at 5 min of stimulation. Center images show Arp2 and BCAP merge staining (red and green) and the bright field with beads conjugated with a negative ligand (BSA) or α-IgM at different time points. The right scheme depicts the area defined as synaptic interface where (B) and (C) were calculated. Arrows highlight the interface of B cells with the bead. (**B**) Pearson coefficient of the colocalization of BCAP with Arp2/3 at the area of the bead presented as dot graph (left) or line graph (right). Graph of data of two independent experiments combined (83 to 62 cells per condition). (**C**) MFI of Arp2 at the synaptic interface. Data of two independent experiments combined (137 to 109 cells per condition). *P* values of less than 0.05 are shown. **P < 0.01, ***P < 0.001, and ****P < 0.0001 by two-way ANOVA with multiple comparisons test. Schematic in (A) created using BioRender.com.

We next investigated whether BCAP is involved in actin reorganization around the antigen due to its ability to recruit actin-regulatory proteins. Immunoprecipitation studies in macrophages showed interaction of BCAP with actin-regulatory proteins such as Flightless-1, gelsolin, and the Arp2/3 complex (*20*). Therefore, as a first approach, we investigated the role of BCAP in the recruitment of the Arp2/3 complex, which is essential for BCR-induced actin reorganization through actin branching and subsequent B cell activation (*22*). B cells were stimulated with α-IgM–coated beads as above, and we stained for BCAP and Arp2, which is part of the Arp2/3 complex. We defined the area of the synaptic interface between the cell and the bead by a rectangle with a width of a quarter of the cell diameter positioned in the middle between the cell and the bead and measured the colocalization of both stains there (Fig. 5A) (*23*). In control cells, we found that BCAP is recruited to the area of contact with the bead, and there is a rapid increase in BCAP-Arp2/3 colocalization at the synaptic interface from 5 to 15 min after stimulation, which returned to basal levels at 30 min. This was specific to BCR activation as we did not see significant colocalization of BCAP and Arp2/3 when we used beads coated with bovine serum albumin (BSA; BCR-negative ligand). We observed minimal BCAP staining in the BCAP KO B cells, and, therefore, colocalization of BCAP with Arp2 was not seen (Fig. 5, A and B). When we measured the Arp2/3 kinetics at the synaptic interface, we found that, in control cells, Arp2/3 staining increased at 5 min but then decreased at later time points. In comparison, BCAP KO B cells showed a delayed arrival of Arp2/3, which remained in that area until later time points (Fig. 5C). This persistence of Arp2/3 on the bead area at 30 min is similar to the persistence of F-actin in the same region shown previously (Fig. 4, H and I). Thus, these data show that the interaction of BCAP with actin regulatory proteins, such as the Arp2/3 complex, is required for the reorganization of actin around the antigen.

### Loss of BCAP leads to defects in antigen-BCR trafficking and propagation of BCR signaling

**Fig. 6.**
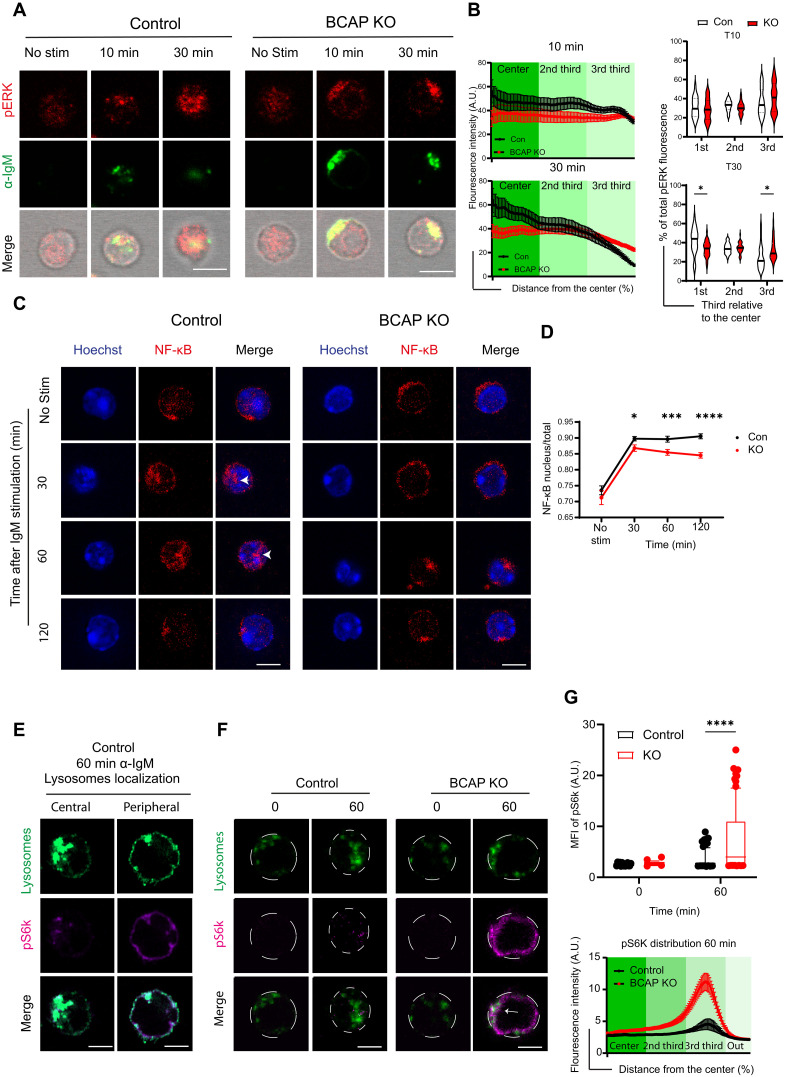
Defects in antigen-BCR complex trafficking in BCAP KO B cells affect the propagation of BCR signaling. (**A**) Representative confocal images of BCAP KO or control splenic B cells resting or activated with α-IgM–biotin (10 μg/ml) for the indicated time points. Cells were fixed and stained with streptavidin-AF488 (green), pERK (red), and the bright field is shown at greyscale. Images are shown as a single plane. Scale bars, 5 μm. (**B**) Radial quantification of normalized pERK fluorescence from BCAP KO and control B cells relative to inner cell radius and violin plots showing the percentage of total fluorescence in the different thirds (61 to 76 cells per condition). Graphs represent one of the two independent experiments yielding similar results. (**C**) Confocal images of BCAP KO or control splenic B cells activated with α-IgM–biotin (10 μg/ml) for the indicated time points. Cells were fixed and stained with streptavidin-AF88 (green) and NF-κB (red). One single plane is presented. Scale bars, 5 μm. (**D**) Image quantification of nuclear NF-κB (defined by a Hoescht mask in the middle of *z*-stack) normalized by NF-κB in the whole cell. Graphs represent two different experiments yielding similar results (*n* = 319 to 242 cells per condition). (**E** and **F**) Confocal plane images of spleen B cells from control and KO mice fixed and stained for lysosomes (green) and pS6K (magenta) in resting (0 min) or activated condition (60 min). Arrow indicates lysosome and pS6k positioned in the periphery. Dashed lines based on the contour of the cell by bright field. Scale bars, 5 μm. (**G**) Quantification of pS6K MFI from images in (F) by box plot (top) or radial distribution of fluorescence at 60 min of α-IgM stimulation (bottom). Representative graph from *n* = 2 experiments yielding similar results (159 to 118 cells per condition). Two-way ANOVA with Šidák’s multiple comparisons test. *P < 0.05, ***P < 0.001, and ****P < 0.0001. No stim, non-stimulated.

Appropriate trafficking of antigen and the BCR is essential for the distribution of antigen-associated signals to distinct cellular compartments. Therefore, we predicted that this disruption in BCR endocytosis and signal organization in BCAP KO B cells would affect the propagation of antigen-induced downstream BCR signaling and activation of transcription factors. We thus used confocal microscopy to evaluate trafficking of antigen-induced BCR signals that depend on their subcellular localization to efficiently propagate the signal. We first evaluated signal propagation using phosphorylated extracellular signal–regulated kinase (pERK) as an example of an antigen-induced signal that signals from vesicles inside the cell (*19*). B cells were stimulated with soluble fluorescent α-IgM for antigenic stimulation and fluorescence of pERK and α-IgM around the cell surface, and the center of the cell was assessed in concentric circles to measure antigen-induced signal propagation. In control B cells, the fluorescence of pERK propagated toward the center of the cell and largely co-localized with antigen at 10 and 30 min. However, in the BCAP KO B cells, pERK and α-IgM both accumulated near the cell boundaries, indicating that the altered spatial organization of BCR and antigen restricts the propagation of antigenic signal (Fig. 6, A and B). To further understand the functional impact of this impaired distribution of antigen-induced BCR signal, we followed signal propagation to the nucleus. Upon BCR stimulation with antigen, transcription factors such as NF-κB are activated and translocate to the nucleus. NF-κB is involved in essential processes of the B cell, such as proliferation, cytokine production, and class switching (*24*). Therefore, we assessed the capacity of the NF-κB signal induced by α-IgM stimulation of BCR to relocate into the nucleus in the BCAP KO cells. Upon α-IgM stimulation of the BCR, in control cells, NF-κB fluorescence increased in the nuclear area delimited by Hoechst staining, indicating NF-κB activation and translocation into the nucleus. In contrast, NF-κB remained near the cell surface in BCAP KO B cells, and there was less NF-κB translocation to the nucleus (Fig. 6, C and D). Thus, impaired BCR and antigen positioning in the BCAP KO B cells impairs antigen-induced signal propagation and, probably, leads to a decrease in transcription factor activity. We predict this is what leads to a decrease in antigen-specific B cell responses in the BCAP KO mice.

To further understand how this accumulation of antigen-induced signals in the periphery in BCAP KO B cells affects other signaling pathways, we assessed the activity of the mammalian target of rapamycin (mTOR)–related kinase S6 kinase (pS6k). Phosphorylation of S6k is mediated by mTOR signaling, which has been linked to lysosomal positioning, with increased mTOR activation seen in peripherally located lysosomes (*25*). We found that, in control cells, lysosomes distribute toward the center; meanwhile, lysosomes accumulated peripherally with the antigen-activated BCR in BCAP KO cells (fig. S7, A and B), and we reasoned that this would lead to an increase in mTOR-mediated pS6K in these cells. B cells were stimulated with α-IgM, and we followed the localization of lysosomes and pS6K. As predicted, in control cells, majority of pS6K fluorescence was localized to peripheral lysosomes at 60 min after stimulation (Fig. 6E). We saw a marked increase in pS6K fluorescence at this time point in KO cells compared to control cells, and this signal was associated with peripheral lysosomes (Fig. 6, F and G). These data confirm that the antigen-induced signals build up in the periphery, unable to traffic toward central areas in BCAP KO cells. This alteration in trafficking appears to be beneficial for optimal activation of proteins such as S6K, whose activation is favored by peripheral lysosomal positioning. This increase in pS6k activity in the BCAP KO cells is dependent on PI3K activation, as we saw a decrease in pS6k activation in BCAP KO cells treated with PI3K inhibitor ZSTK474 (fig. S7C). Increased mTOR-related S6 activity could have consequences in class switching (*26*) and consistent with this hypothesis, we observed an increased percentage of IgG plasma cells versus IgM plasma cells in our in vitro plasma cell differentiation assay with α-IgM stimulation of BCR (fig. S7, D and E). This differentiation is antigen unspecific, highlighting that differences seen in vivo (Figs. 1J and 2J) are due to an antigen-specific recognition. In summary, BCAP plays an important role in the distribution of BCR-derived antigen signals from the cell surface to central areas, and alterations in this activity can have profound impacts on the outcome of BCR signaling.

### Defects in antigen processing due to loss of BCAP affect antigen presentation by B cells

**Fig. 7.**
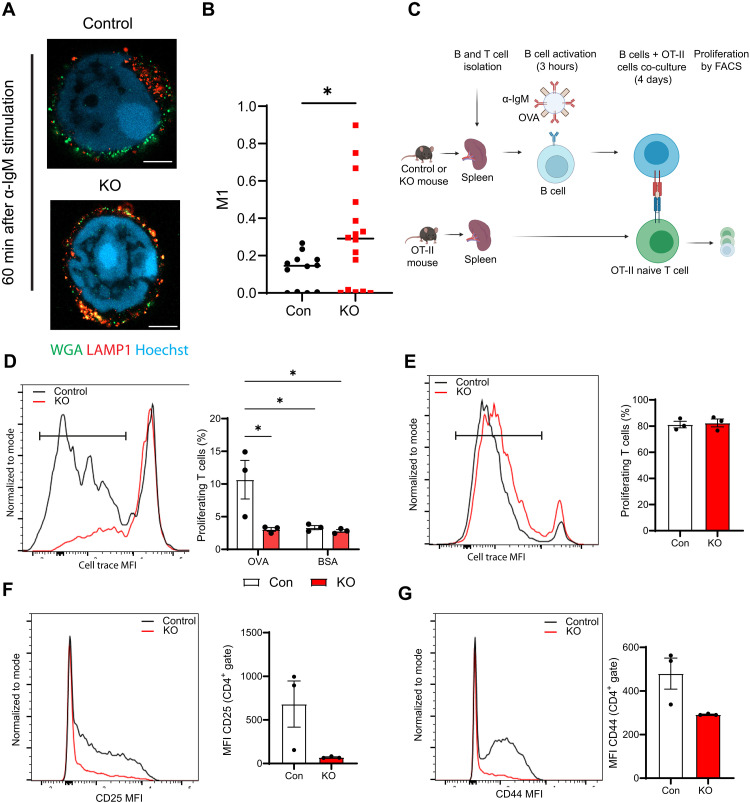
Defects in antigen-BCR complex trafficking in BCAP KO B cells impact antigen presentation. (**A**) Expansion microscopy of control and BCAP KO cells after 60 min of IgM stimulation. Cells were fixed and stained with WGA (green), LAMP1 (red), and Hoechst (Blue). Images are shown as a single plane. Scale bars, 5 μm. (**B**) Manders’ colocalization between WGA and LAMP1 performed in one plane; 12 to 16 cells from two independent experiments combined are presented. (**C**) Scheme for antigen presentation assay. (**D**) Representative graph of antigen presentation assay of control or BCAP KO B cells. Levels of proliferation of T cells were quantified following cell trace dilution by flow cytometry. (**E**) Representative graph of peptide controls for cells used in antigen presentation assays. (**F** and **G**) Levels of CD25 and CD44 on the surface of T cells activated by control or BCAP KO B cells. Graph shows means and SEM of three technical replicates and representative of three independent experiments yielding similar results. One-way ANOVA with multiple comparisons (D) or Mann-Whitney *U* test [(B) and (E) to (G)] were calculated. *P < 0.05. (C) created using BioRender.com.

We next sought to further establish the functional impact of this role of BCAP in positioning and distribution of antigen derived signals in B cells. One consequence of the defect in trafficking of antigens and associated signals toward the central areas in BCAP KO B cells is that the antigens may not have access to central compartments such as lysosomes and multivesicular bodies important for antigen processing and presentation (*27*). Moreover, the location of lysosomes has been shown to be important in B cells for antigen extraction, processing, and presentation (*28*), and the arrested traffic of lysosomes in the BCAP KO cells (fig. S7A) further predicted effects on antigen processing. Therefore, we analyzed the lysosomal compartments in the BCAP KO B cells in more detail. We did not find major defects in colocalization of the antigen with the lysosomal marker LAMP1 in BCAP KO B cells compared to those in control B cells (fig. S7A), and we further evaluated the structure of the peripheral lysosomes in the BCAP KO B cells. To aid this evaluation, we used expansion microscopy, which allows us to see intracellular structures with better resolution. We stained control and BCAP KO cells after 60 min of α-IgM stimulation with LAMP1 as a marker of the lysosomes and WGA as a marker of the plasma membrane and analyzed lysosomal accumulation. In control cells, we observed that lysosomes distributed both peripherally and near the perinucleus, but, unexpectedly, in the KO cells, not only did we observe peripheral lysosomes, but there was also colocalization of LAMP1 with WGA, suggesting an exacerbated lysosomal secretion. Lysosome secretion is not normally associated with soluble antigens; therefore, these findings suggest an abnormal lysosomal reaction to defective antigen internalization in the BCAP KO B cells (Fig. 7, A and B) (*29*). On the basis of these changes in lysosomal localization and secretion in the BCAP KO B cells, we evaluated how the loss of BCAP affects antigen processing and presentation by B cells to T cells. We performed an antigen presentation assay that we have previously used to study antigen extraction and processing by B cells, using beads coated with α-IgM and OVA (*28*, *30*). We incubated spleen B cells from control or BCAP KO mice for 3 hours with latex beads incorporating α-IgM and OVA. B cells were then incubated with T cells from OT-II mice, which recognize OVA peptide. The capacity of B cells to present the major histocompatibility complex class II (MHC-II)–complexed OVA peptide derived from the beads to naïve T cells was then measured by monitoring the proliferation of T cells using cell trace dilution (Fig. 7C). OT-II T cells cultured with control B cells incubated with IgM-OVA beads showed increased proliferation as seen by dilution of cell trace dye, indicating efficient presentation of Ova antigen by B cells to T cells. However, T cells incubated with the BCAP KO B cells showed limited cell trace dilution (Fig. 7D), indicating defects in antigen processing and presentation. To confirm that we were measuring T cell proliferation induced by antigen extracted and processed through the BCR, B cells were also incubated with beads coated with α-IgM and irrelevant antigen BSA. Cultures with both control and KO B cells did not lead to T cell proliferation with α-IgM–BSA beads, confirming that T cell proliferation was due to BCR-mediated presentation of OVA peptide derived from the beads (Fig. 7D).

BCAP KO B cell cultures did not show defects in inducing proliferation of OT-II T cells when antigen in the form of OVA peptide was provided to B cells rather than α-IgM–OVA beads (Fig. 7E). This further confirms defects in antigen processing and presentation in BCAP KO cells and not alterations in MHC-II or costimulatory molecules on the cell surface. We also measured the capacity of these B cells to activate T cells, measuring the surface markers CD25 and CD44 as readouts of T cell activation. As expected, BCAP KO B cell cultures showed reduced capacity to induce CD25 and CD44 expression on T cells (Fig. 7, F and G) compared to cultures with control B cells. Together, these data show that the function of BCAP in regulating antigen positioning and distribution inside the B cell is essential for optimal processing and presentation of antigens to T cells.

## DISCUSSION

BCAP was first discovered in B cells as an adaptor connecting PI3K to the BCR, and previous studies have shown a role for BCAP in B cell development and antibody responses. However, the mechanisms by which BCAP regulates BCR signaling and the B cell–intrinsic role for BCAP in antibody responses in vivo were not established. Here, we describe a previously unidentified B cell–intrinsic role for BCAP in coordinating antigen endocytosis and processing of antigen associated signals during early B cell activation. Our data show that BCAP is required for antigen endocytosis and processing because of its ability to coordinate actin dynamics at the point of contact between the cell and the antigen. Loss of BCAP from B cells thus leads to defects in actin clearance during antigen endocytosis and subsequent intracellular positioning of antigens. This defect in the antigen trafficking in BCAP KO B cells has multiple consequences for B cell function, including a defect in the propagation of antigenic signal, a decrease in B cell antigen presentation capacity, and, consequently, a decrease in antigen-specific B cell response, particularly for complex antigens such as viral particles.

Antigen binding to the BCR activates both BCR signaling and endocytosis, which, in turn, regulate each other. Impaired BCR endocytosis by mutations in BCR co-receptors, Igα or Igβ chains, leads to increased BCR signaling and an enhanced response to the T-independent antigen as well as slightly decreased response to the T-dependent antigen NP-CGG (*31*). Endocytosis of the BCR is also essential for proper compartmentalization of BCR signaling and regulation of downstream signal (*19*). In addition, the endocytosis of the BCR is vital for the trafficking of the TLR ligand associated with the antigen to appropriate compartments for interaction with their receptors (*32*). Our data add to these findings by revealing how changes in BCR-antigen endocytosis impact the outcome of B cell response. BCAP KO B cells show a notable loss of proliferation after IgM stimulation of BCR, which is associated with an equally notable increase in global antigen-derived signals that accumulate in the periphery of the cell. This change in the distribution of antigen-derived signals affects multiple aspects of the B cell response. First, antigen-induced BCR signals are not properly propagated, resulting in decreases in the downstream signal transduction and the translocation of transcription factors such as NF-κB to the nucleus. Thus, B cells cannot expand in response to antigenic signals, and BCAP KO mice showed a decrease in antigen-specific GC B cells and switched memory B cells necessary for adequate humoral responses after immunization. Second, because of the inability to distribute antigens and associated signals in the central areas, the ability of B cells to present antigens to T cells is compromised. Antigen internalization in B cells facilitates trafficking through endosomal compartments to specialized late endosomal compartments rich in MHC-II (*27*) and other enzymes required for efficient antigen processing. In the BCAP KO B cells, the accumulation of lysosomes in the peripheral areas and lysosomal secretion in response to soluble antigens could represent compensation for impaired BCR endocytosis, and this likely affects lysosomal functions necessary for antigen presentation. The capacity of peripheral lysosomes seen in the BCAP KO cells to process antigen is unclear, and we predict that changes in these lysosomes related to the machinery needed for optimal antigen presentation, such as MHC-II molecule, proteasomal content, or acidification, result in less antigen presentation capacity of the BCAP KO B cells. More studies are necessary to investigate further details of the changes in lysosomes in the absence of BCAP. In addition, in the context of VLP immunization, proper processing of the VLP would be required for the delivery of the ssRNA incorporated in the VLP to endosomal compartments containing TLR7. This will be defective in BCAP KO B cells; therefore, these B cells will not receive adequate TLR7 signals due to defects in BCR endocytosis. However, a role for BCAP processing of TLR signals cannot be excluded. BCR-independent effects of BCAP in TLR signaling have been described in myeloid cells (*5*–*7*, *15*, *33*, *34*) and could be linked to decreased proliferation in response to TLR7 or TLR9 ligands stimulation seen in vitro (Fig. 3, E and F).

The peripheral localization of antigens in the BCAP KO B cells and subsequent peripheral lysosome localization could have advantages, such as in activating mTOR. Studies in epithelial cells have shown that redistribution of lysosomes to the cell periphery by overexpression of kinesins KIF1Bβ, KIF2, or Arl8 increases activation of mTOR pathway (*25*). The peripheral lysosomal positioning could favor lysosomal capture of amino acids and growth factors, helping to activate the mTOR pathway (*25*). Deason *et al.* (*9*) have shown a decrease in mTOR activation in BCAP KO T cells through interleukin-1R (IL-1R)–PI3K pathway activation but not by TCR activation. In contrast our findings of peripheral lysosomal localization indicate that there could be higher mTOR activation after BCR engagement in the BCAP KO B cells. BCR engagement promotes the recruitment of polarity proteins such as aPKC/Cdc42 and Par3 that play a critical role in repositioning the centrosome and lysosomes near the BCR-antigen domain (*35*). Lysosomes are retained in the peripheral area in the BCAP KO B cells, probably as a consequence of defects in actin cortex remodeling and endocytic internalization mediated by BCAP interacting proteins such as Arp2/3 (*21*, *36*, *37*). BCAP KO B cells show an increase in phosphorylated S6 kinase after BCR engagement, which is associated with peripheral lysosomes. This pS6k activation is still dependent on PI3K activation, as the increased pS6k response to the BCR stimulation is lost when we use the class I PI3K inhibitor ZSTK474 (fig. S7A). These data also confirm that, as previously described, PI3K activation is intact in BCAP KO cells (*2*) and is still functional in activating mTOR pathway via the peripheral lysosomes. We predict that the increase in class-switched B cells and activated GC-like B cells seen in the BCAP KO in vitro cultures likely reflects the impact of increased mTOR-related pS6k signaling (Fig. 3C and fig. S7D). This increase in activation of mTOR-related pathways may also indicate that loss of BCAP mainly affects primary B cell responses, as, over time, BCAP KO B cells could overcome issues of expansion by increasing their ability to class switch. This agrees with previous studies, which have shown that loss of BCAP does not affect secondary immune responses (*2*).

Previous studies in other immune cell populations have shown a role for BCAP in regulating signaling through the TLR or cytokine receptor signaling (*5*, *6*, *9*, *34*) via PI3K activation. In B cells, the mechanistic details related to PI3K are less clear, as studies showing defects in Ca^2+^ mobilization through the BCR are not associated with defects in PI3K activation (*2*). Moreover, besides PI3K, we have shown that BCAP interacts with several F-actin–regulatory proteins in macrophages, such as the actin capping protein Flightless-1, the actin bundling protein L-plastin, the actin capping and severing protein gelsolin, and members of the Arp2/3 complex (*21*). Our studies indicate that the primary role of BCAP in BCR signaling is to recruit resources such as actin-regulatory proteins and possibly activated PI3K around the antigen during early antigen encounter by B cells. BCR engagement is connected to actin polymerization and treatments of cells with inhibitors of actin cytoskeleton blocked redistribution of BCR from the plasma membrane into intracellular endosomes (*20*), similar to what we have observed in the BCAP KO B cells. We observed that, in control cells, there is F-actin clearance around the antigen following BCR engagement. This F-actin clearance is not observed in BCAP-deficient B cells, implying that BCAP may act as a polarity cue to attract the centrosome, which, upon BCR engagement, concentrates many actin regulators to the area of the antigen and facilitates secondary F-actin clearance (*38*). Moreover, our data show that the ability of BCAP to bind to actin regulatory proteins such as the Arp2/3 complex is important for its ability to regulate actin reorganization events for antigen endocytosis. It is likely that BCAP interacts with other actin-related proteins that are involved in actin clearance. Moreover, BCAP signaling orchestration could differ between soluble and immobilized antigen as Arp2/3 also is implied in immobilized antigen signaling amplification, but not soluble (*22*). Future work is required to delineate the exact mechanisms by which BCAP orchestrates actin dynamics for BCR endocytosis and signal positioning.

One limitation of our study is we do not know whether the BCAP regulation seen in IgM stimulated cells also extends to other Ig isotypes in B cells. Because a previous study showed that IgG^+^ B cells outcompete IgM^+^ B cells selection in the GC (*39*), we can speculate that this role of BCAP has different relevance in distinct B cells isotype populations. Although previous studies showed that secondary responses were not affected in the BCAP KO mice (*2*), these were performed in global BCAP KO mice; therefore, they could reflect the contribution of BCAP on other immune cell populations. It is also possible that memory B cells internalize antigens via mechanisms that are different from naïve B cells, and, thus, BCAP could be more essential for antigen processing by naïve B cells, expressing the IgM BCR (*40*). Moreover, memory B cells are reliant on receptors such as TLRs for activation; therefore, they may be less dependent on BCAP-mediated internalization of antigens through the BCR. Further studies are necessary to confirm whether these effects of BCAP in antigen processing are relevant for memory B cells and secondary responses.

Overall, this work allows us to connect how antigen signals regulate the outcome of B cell response and how small changes in intracellular localization of antigens can have profound effects in in vivo immune response. These results also highlight the potential of modulating intracellular trafficking mechanisms to promote distinct outcomes of immune responses in the context of autoimmunity or antiviral immunity.

## MATERIALS AND METHODS

### Study design

This study aimed to investigate how BCAP regulates BCR responses to complex antigens that exert their response stimulating BCR and TLR receptors. We perform in vivo and in vitro experiments to analyzing B cells and their antibody production in response to different kind of antigens. We use mice models global and conditional KO for BCAP to measure the global humoral response by flow cytometry, enzyme-linked immunosorbent assay, and ELISpot. We isolated B cells from WT and KO mice to study intracellular mechanisms by microscopy and biochemical techniques. Biological and technical replicates were used to validate the findings, and experiments were performed at least twice unless specified otherwise.

### Mice

Wild-type C57BL/6J mice were bred at the animal facility at Seattle Children’s Research Institute or Benaroya Research Institute or purchased from the Jackson Laboratory. BCAP KO mice lacking the *Pik3ap1* gene were backcrossed to C57BL/6J mice and were previously described (*3*). The animals were bred and maintained on the C57BL/6 background. BCAP^fl/fl^ mice were previously described (*9*) and crossed with CD19 cre transgenic mice to generate conditional KO (*Cd19^cre/+^Pik3ap1^fl/fl^*) mice. Littermates with a single *Cd19^cre^* allele were used as *Cd19^cre/+^Pik3ap1^+/+^* controls. OT-II mice were purchased from The Jackson Laboratory. All mice were housed under specific pathogen–free conditions at Benaroya Research Institute or Seattle Children’s Research Institute. All animal experiments were performed under appropriate licenses and institutional review within local and national guidelines for animal care. Both sexes of mice between the ages of 8 and 21 weeks old were used in this study.

### Antibody and reagents

Anti-mouse antibodies used for flow cytometry included the following: B220 [phycoerythrin (PE), catalog no. 553090; BUV395, catalog no. 563793], CD24 [fluorescein isothiocyanate (FITC), catalog no. 553261], CD19 (BUV737, catalog no. 612781), IgD (BV786, catalog no. 563618), and IgG1 [PE-Cyanine 7 (Cy7), catalog no. 550083] were purchased from BD Biosciences. CD4 (FITC, catalog no. 100406), CD21 (BV421, catalog no. 123414), CD23 (PE-Cy7, catalog no. 101614), CD25 (PE, catalog no. 102008), CD138 (BV421, catalog no. 142523), Fas (BV605, catalog no. 152612), IgM (PE, catalog no. 406508), CD38 [allophycocyanin (APC)–Cy7, catalog no. 102728], CD44 (BV605, catalog no. 103047), IgM (PerCP/Cy5.5, catalog no. 406512), B220 (FITC, catalog no. 103206; PE-Cy7, catalog no. 103222), and Live/Dead ZombieRed (catalog no. 77475) were purchased from BioLegend. IRF4 (PE-Cy7, catalog no. 25-9858-82), IgG [Alexa Fluor 488 (AF488), catalog no. A11001], and Cell Trace (Far Red, catalog no. C34572) were purchased from Invitrogen. IgG2c (FITC, catalog no. 1079-02) and anti-mouse IgM biotinylated (catalog no. 1021-08) were purchased from SouthernBiotech.

BCAP antibody was produced in-house (4L8E6, purified from hybridoma) and previously described (*8*). Antibodies used for Western blot included anti–β-actin (AC-15, catalog no. L7543) (Sigma-Aldrich), anti-pTyr (4G10, catalog no. 05-321) (EMD Millipore), and horseradish peroxidase–linked antibody anti-rabbit (catalog no. 7074S) purchased from Cell Signaling Technology.

Antibodies for microscopy included the following: anti–NF-κB p65 (D14E12, catalog no. 8242), pERK (20G11, catalog no. 4376S), Rab5 (C8B1, catalog no. 3547S), and pS6k (AF647 conjugated, clone D57.2.2E, catalog no. 4851) from Cell Signaling Technology; anti-pTyr AF488 conjugated (catalog no. 309306) from BioLegend; and LAMP1 antibody (catalog no. BDB5537) purchased from Thermo Fisher Scientific. The following secondary antibodies were used: Alexa Fluor 488–, Alexa Fluor 568 Cy3–, and Alexa Fluor 647–conjugated F(ab′)2 donkey anti-rat and Cy3-conjugated F(ab′)2 donkey anti-rabbit (1:500; Jackson ImmunoResearch, West Grove, PA, USA) and Alexa Fluor 488–conjugated goat anti-rabbit (Molecular Probes, Invitrogen, Eugene, OR, USA 1:200). F-actin was stained using Alexa Fluor 546– or Alexa Fluor 647– conjugated phalloidin (1:400; Life Technologies, Carlsbad, CA, USA, no. A22287). Streptavidin AF488 conjugated (S11223) was purchased from Invitrogen.

Inactivated H1N1 PR/8 was purchased from Charles River Laboratories. Vaccine-grade TLR7 ligand adjuvant imiquimod-SE was from the Infectious Disease Research Institute. Type B CpG ODN 2006, ODN 2925, and R848 were purchased from InvivoGen. ZSTK-474 PI3K inhibitor was purchased from Selleck Chemicals. All VLP reagents were provided by B. Hou (Chinese Academy of Sciences, Beijing, China). Latex NH2-beads of 3 μm and Streptavidin Fluoresbrite YG Microspheres of 1 μm were purchased from PolyScience.

### Flow cytometry and cell sorting

Cells were harvested in phosphate-buffered saline (PBS)/0.5% BSA/2 mM EDTA. Splenocytes or BM cells were depleted of red blood cells (ACK Lysis Buffer; Gibco, Thermo Fisher Scientific). Single-cell suspensions were blocked with Fc Block (BD Biosciences) and stained with fluorochrome-tagged antibodies for surface markers (1:200 dilution) at 4°C for 30 min. For detection of Qβ-specific cells, cells were stained with Alexa Fluor 647–labeled Qβ-VLPs together with the antibodies for surface markers. Antibody isotype detection on plasma cells differentiation was performed by permeabilization of the cells with True-Nuclear Transcription Factor Buffer Set (BioLegend) after surface staining. Samples were acquired using an LSR Fortessa flow cytometer (BD Biosciences) and analyzed by FlowJo software (Tree Star Inc.). For sorting of spleen follicular cells, after B cell enrichment with the negative selection cocktail (Stem Cell Technologies), cells were labeled with anti–B220-PE, anti–CD21-BV421, anti–CD23-PE-Cy7, anti–CD24-FITC and then sorted with FACSAria (BD Biosciences).

### In vitro proliferation assay

B cells from the spleens were sorted as follicular, marginal zone, or transitional cells. Sorted cells were plated in complete RPMI 1640 [10% fetal bovine serum (FBS), 2 mM glutamine, penicillin (100 U ml^−1^) and streptomycin (100 μg ml^−1^), and 50 μM 2-β-mercaptoethanol] at a density of 3 × 10^4^ cells per well on 96-well plate and treated with different stimuli: CpG-C (2 μM), R848 (5 μg ml^−1^), and α-IgM F(ab′)2 (10 μg ml^−1^). After 48 hours, cells were pulsed with [3H]-thymidine at 1 μCi per well for 18 hours before harvest; thymidine incorporation was determined by liquid scintillation spectrometry.

### Immunization

Mice were immunized intraperitoneally with NP-haptenated chicken γ globulin (NP-CG) (Biosearch Technologies), 50 μg per mouse in combination with alum (1:1), or 10 μg of clinical-grade TLR7 ligand adjuvant imiquimod-SE (Infectious Disease Research Institute). For VLP experiments, VLP derived from Qβ bacteriophages were provided by Baidong Hou (Institute of Biophysics, Chinese Academy of Sciences, Beijing, China). The production of these particles has been described previously (*13*). Mice were immunized intraperitoneally with 2 μg of VLPs, and serum was collected at indicated time points by submandibular bleeds. For studies with intact virus, mice were immunized intraperitoneally with 10 μg of inactivated H1N1 PR/8 influenza virus (Charles River Laboratories).

### Enzyme-linked immunosorbent assay

Immulon 2HB plates were coated with inactivated PR8 (1 mg/ml), VLP (1.5 mg/ml), and NP-BSA (1 mg/ml) (*13*). They were blocked for 2 hours at 37°C with block solution (2% BSA, 2% fetal calf serum, 0.1% Tween 20, and 0.02% sodium azide in PBS). Sera was diluted at 1:200 in 50% block, plated, and incubated at 4°C. overnight. Subclass-specific antibodies were detected using goat anti-mouse alkaline phosphatase-conjugated IgM, IgG, and IgG2c antibodies (SouthernBiotech) (1:1000 dilution). Secondary antibody was detected with disodium p-nitrophenyl phosphate substrate (Thermo Fisher Scientific or Sigma-Aldrich) and the absorbance read at 405 nm.

### Enzyme-linked immunosorbent spot

To detect antigen-specific long-lived plasma cells in the BM, 96-well filter plates (Merck Millipore) were coated overnight with antigen: VLP (2 μg/ml), NP-BSA (10 μg/ml), or Ig (H+L) (SouthernBiotech) in PBS at 4°C. After blocking with RPMI 1640 with 10% FBS for 1 hour, twofold dilutions of BM single-cell suspension were added to the filter plates starting with 2 × 106 cells per well in RPMI 1640 with 10% FBS and incubated for 12 hours at 37°C. Cells were plated at 20 million cells per milliliter at initial concentration. Plates were washed with PBS and incubated with Alkaline Phosphatase (AP)–conjugated anti-mouse IgM, total IgG, or IgG2c (SouthernBiotech) and developed with 5-bromo-4-chloro-3-indolyl phosphate (BCIP)/nitro blue tetrazolium (NBT) alkaline phosphate substrate kit (Vector Laboratories). Plates were washed with PBS, and positive cells were counted in each well.

### Plasma differentiation assay

Spleen cells were isolated in complete Iscove’s Modified Dulbecco’s Medium (IMDM) medium, and B cells were isolated using negative magnetic bead separation EasySep Mouse B cell isolation kit by STEMCELL Technologies following their instructions in the kit. Cells were counted with the Muse cell counter and stimulated with either non-TLR conditions MCD40L (100 ng/ml; AdipoGen) and IgM (1.3 mg/ml; Jackson ImmunoResearch) or TLR conditions CpG B (1 mg/ml; InvivoGen). B cells (10^6^ cells) were incubated in 24-well plate for 2 days for activation and then seeded over irradiated 40LB cells (2 × 10^4^ cells) expressing CD40L and BAFF (*17*) for 3 days in IL-6 (10 ng/ml; PeproTech) and IL-21 (40 ng/μl, PeproTech) for the non-TLR conditions and R848 (1 mg/ml; InvivoGen) and IL-21 for the TLR conditions. All cells were then harvested, washed, and replated onto the original feeders with IL-21 for 3 days.

### Western blot

Western blot was prepared as previously described (*41*). Briefly, cells were lysed in hypotonic nuclear extraction buffer [1 M Hepes (pH 7.5), 5 M NaCl, 0.5 M EDTA (pH 8), 50% glycerol, 10% Igepal (Sigma-Aldrich), and 10% Triton X-100 (Sigma-Aldrich)] supplemented with protease inhibitor cocktail (Pierce) for 10 min followed by centrifugation at 1500*g* for 5 min at 4°C to pellet the nuclei. Supernatant was used as cytoplasmic extract. Proteins were quantified by Bicinchoninic acid (BCA) assay (Pierce), separated by electrophoresis using NuPage-Bis-Tris gels (Invitrogen), and blotted onto polyvinylidene difluoride membranes. Nonspecific binding was blocked with 5% BSA in TBS-Tween (0.1%), followed by incubation with primary antibodies (1:1000 dilution) overnight at 4°C and secondary antibody horseradish peroxidase–conjugated antibodies (1:2000 dilution) for 1 hour at room temperature (RT). Membranes were washed thoroughly with TBS-Tween (0.1%) after antibody incubations and developed using enhanced chemiluminescence (ECL) reagents (Merck Millipore).

### qPCR analysis

Primary B cells from control and conditional KO mice were isolated and left unstimulated. RNA was prepared using a QIAGEN RNeasy mini kit and converted into cDNA by reverse transcription (Applied Biosystems). Real-time PCR was performed with SYBR Green (Applied Biosystems) using the following primers: *Tlr9*: [forward (fw)] ACTCCGACTTCGTCCACCT and [reverse (rev)] GGCTCAATGGTCATGTGGCA; and *Tlr7*: (fw) GGCTGAACCATCTGGAAGAA and (rev) TAAGCTGGATGGCAGATCCT.

### Antigen internalization by flow cytometry

Protocol for antigen internalization was adapted from Hernández-Pérez *et al.* (*42*). Splenocytes were stained on ice for 10 min with α-IgM conjugated to biotin (SouthernBiotech) in buffer (PBS/0.5% BSA/2 mM EDTA) buffer. Cells were incubated at 37°C and 5% CO_2_ at different time points. For time 0, the samples were kept on ice all the time after incubation and stained with streptavidin-488 for 20 min, before being washed and analyzed at LSR Fortessa (BD Biosciences).

### Preparation of antigen-coated beads and antigen-coated coverslip

Antigen-coated beads were prepared as previously described (*28*). Briefly, ∼2 × 10^7^ 3-μm latex NH2-beads (PolyScience) were activated with 8% glutaraldehyde for 4 hours at RT. Beads were washed with PBS and incubated overnight at 4°C with different ligands: F(ab′)2 goat anti-mouse IgM (α-IgM; 100 μg/ml) or BSA (100 μg/ml). For antigen presentation assays, beads were coated with α-IgM or BSA plus OVA (100 μg/ml). For fluorescent bead assays, 5 μl of Streptavidin Fluoresbrite YG Microspheres were conjugated with anti-mouse IgM (100 μg/ml) biotinylated at 4°C overnight. Antigen coverslips used to analyze the synaptic interface were coated with BCR ligand^+^ overnight at 4°C in PBS at 100 μg/ml.

### Antigen presentation

Antigen presentation assays were adapted from previous works (*28*, *30*). Briefly, isolated primary B cells were incubated with either OVA–α-IgM– or BSA–α-IgM–coated beads or OVA peptide (323-339, Invitrogen; 10 mg/ml) for 3 hours. B cells were then incubated with isolated naïve T cells from OT-II mice spleen previously stained with cell trace in a 2:1 ratio for 4 days. Proliferation of OT-II cells was measured by cell trace dilution by flow cytometry. T cell activation was measured by assessing CD25 and CD44 on the cell surface using flow cytometry.

### Immunofluorescence and microscopy

For analysis of the synaptic plane, IIA1.6 B cells were plated onto BCR ligand^+^–coated glass coverslips for different time points and incubated at 37°C, as previously described (*35*). Cells were stimulated with the BCR ligand–coated beads at a 1:1 ratio (cells to beads) or α-IgM–biotin for the indicated times at 37°C, plated on poly-l-lysine-coated coverslips and fixed in 4% paraformaldehyde for 10 min at RT. Fixed cells were incubated for 60 min with primary antibodies and 60 min with secondary antibodies in PBS-BSA-saponin (1×/0.2%/0.05%). Coverslips were mounted onto slides using Fluoromount G (Electron Microscopy Sciences, Hatfield, PA, USA). Cells were imaged on a 63× oil objectives (aperture of 1.4) on Leica TCS SP5 confocal microscope (Leica, Wetzlar, Germany) or Zeiss 780 Confocal Ayriscan (100×/1.45, oil) in Fred Hutch or Zeiss 900 (63×/1.4, oil) confocal on Microscopy and Histopathology CoLab at Seattle Children’s Research Institute.

### Expansion microscopy

Expansion microscopy protocol was adapted from Königshausen *et al.* (*43*). Shortly after regular immunofluorescence protocol, cells were leave overnight with Acryloyl-X, SE (0.1 mg/ml; Invitrogen) in 150 mM NaHCO_3_. Then, coverslips were placed in a glass slide between two stacks of 1.0 and 1.5 rectangular coverslip stripes. Gelling solution [sodium acrylate (86 mg/ml), acrylamide (25 mg/ml), *N*,*N*′-methylenebisacrylamide (1 mg/ml), NaCl (117 mg/ml), ammonium persulfate (APS) (2 mg/ml), and tetramethylethylenediamine (TEMED) (2 mg/ml) (all from Sigma-Aldrich) in 1× PBS (Gibco)] was applied to the coverslip and left them polymerizing 1 hour at 37°C. Gels were stored in 1× PBS and digested the next day with 0.05% Triton X-100 (Thermo Fisher Scientific), 1 mM EDTA (Gibco), 50 mM tris (Sigma-Aldrich), NaCl (4.67 g/ml), ddH_2_O, and proteinase K 800 (U/ml) (New England Biolabs) 1 hour at 37°C. Then, gels were expanded with four incubations with ddH_2_O 10 min at RT, and, then, appropriate pieces of gels were placed in a six-well glass bottom plate previously incubated with poly-l-lysine 20 min at RT and imaged in an inverted Zeiss 980 (63×/1.4, oil) confocal microscope.

### Image analysis

Image analysis was performed using Fiji software (*44*). The pERK distribution or α-IgM distribution (integral radial pixel intensities) was determined using immunofluorescence imaging, and clock scan analysis was performed (*45*). Cell borders were selected from bright field and defined with the ImageJ ROI tool for at least 30 cells. All ROIs were then analyzed using the clock scan protocol implemented as a Fiji ImageJ plugin. The intensities were measured separately for the pERK and α-IgM channels. The means ± SEM of all nuclei were plotted for each % of the radius. The Kolmogorov-Smirnov test was used to detect significant differences.

Measuring of NF-κB translocation was done by CellProfiler software (*46*). Nucleus were segmented IdentifyPrimaryObject module by the hoescht stain into the median plane of every cell. Cells was segmented by IdentifySecondaryObject module F-actin fluorescence in the same planes. Nuclear translocation of NF-κB for each cell was calculated as the mean fluorescence intensity (MFI) of NF-κB in the nucleus mask normalized by the NF-κB MFI of total cell. F-actin three-dimensional projections were made using Imaris software (Oxford instrument). For quantification of Rab5 and Α-IgM colocalization, Pearson’s correlation was calculated using Coloc2 plugin from Fiji software. For BCAP and Arp2/3 colocalization and MFI measurement, we use Fiji to analyze a synaptic area previously described (*23*). Actin reorganization was performed with the Quadrant picking plugin in Fiji and calculating the percentage of actin in every quadrant of the total actin on the bead.

### Statistical analysis

Data were collected from two to three independent experiments and reported as means ± SEM. Statistical analysis was performed using Student’s *t* test or one- or two-way analysis of variance (ANOVA) and post hoc analysis for multiple comparisons (specified in the figure legends) using Prism (GraphPad Software, San Diego, CA, USA). The *P* values were computed using different tests, as indicated in the figure legends: *0.01 < *P* < 0.05, **0.001 < *P* < 0.01; ***P < 0.001; ns, not significant.
