# Supplementary material for: B cell adapter for PI 3-kinase (BCAP) coordinates antigen internalization and trafficking through the B cell receptor
Source: Sci Adv. 2024 Nov 15;10(46):eadp1747. doi: 10.1126/sciadv.adp1747 (PMC11566990; doi:10.1126/sciadv.adp1747)
Supplement: Supplementary file 1 — Figs. S1 to S7 [file sciadv.adp1747_sm.pdf]

Science Advances
AAAS

Supplementary Materials for

B cell adapter for PI 3-kinase (BCAP) coordinates antigen internalization and trafficking through the B cell receptor

Jonathan Lagos *et al.*

Corresponding author: Mridu Acharya, mridu.acharya@seattlechildrens.org

Sci. Adv. **10**, eadp1747 (2024)
DOI: 10.1126/sciadv.adp1747

This PDF file includes:

Figs. S1 to S7

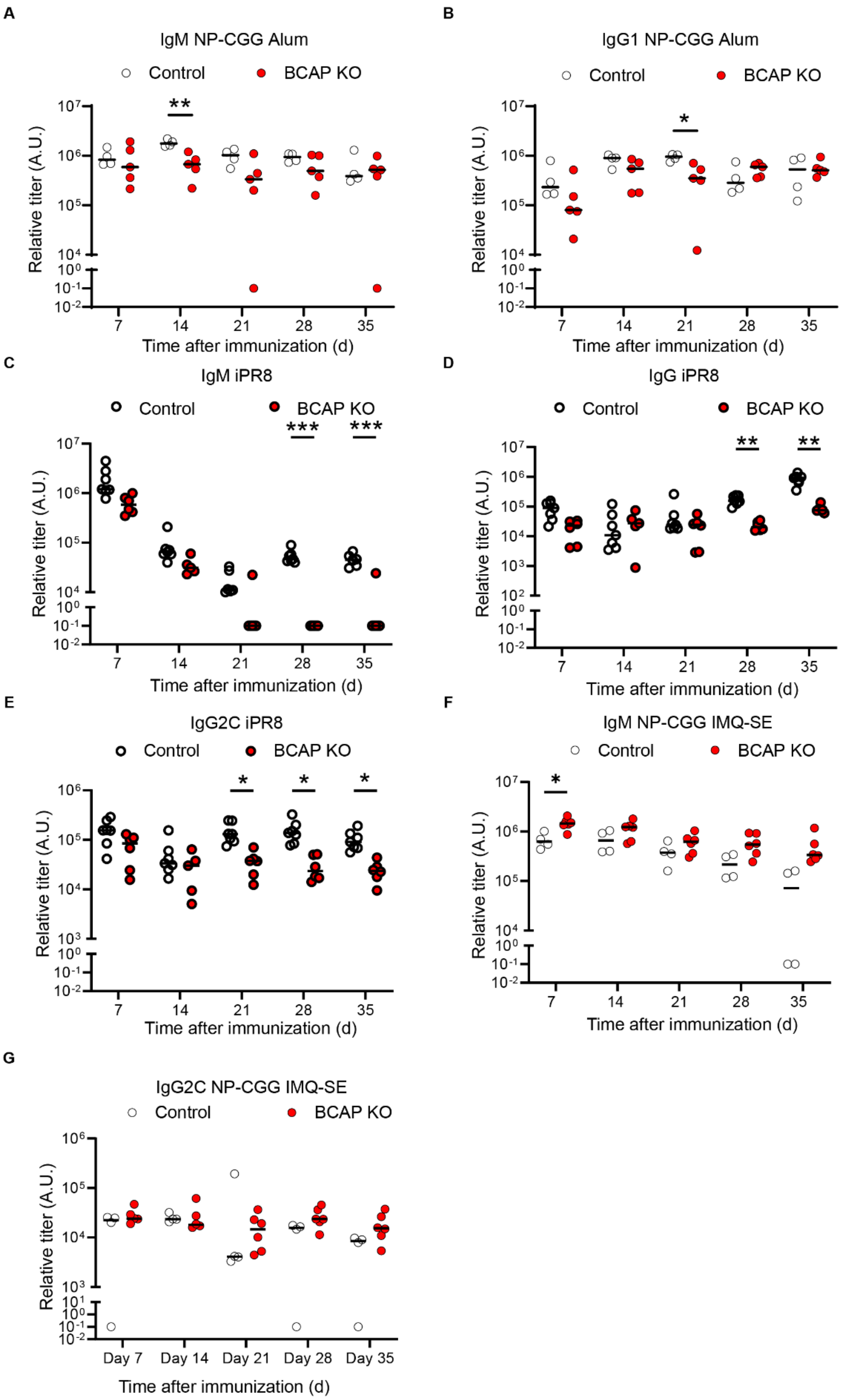
A
IgM NP-CGG Alum
Control
BCAP KO
**
Relative titer (A.U.)
Time after immunization (d)
B
IgG1 NP-CGG Alum
Control
BCAP KO
*
Relative titer (A.U.)
Time after immunization (d)
C
IgM iPR8
Control
BCAP KO
***
***
Relative titer (A.U.)
Time after immunization (d)
D
IgG iPR8
Control
BCAP KO
**
**
Relative titer (A.U.)
Time after immunization (d)
E
IgG2C iPR8
Control
BCAP KO
*
*
*
Relative titer (A.U.)
Time after immunization (d)
F
IgM NP-CGG IMQ-SE
Control
BCAP KO
*
Relative titer (A.U.)
Time after immunization (d)
G
IgG2C NP-CGG IMQ-SE
Control
BCAP KO
Relative titer (A.U.)
Day 7
Day 14
Day 21
Day 28
Day 35
Time after immunization (d)

Fig. S1. Levels of antigen-specific antibodies after different immunizations.

(A-B) Serum anti-NP-CGG IgM or IgG1 antibody titers in control and BCAP KO mice, immunized with 50 µg of NP-CGG in combination with alum (1:1) per mouse at 7,14,21,28, and 35 days after immunization. **(C-E)** Serum anti-iPR8 IgM or IgG or IgG2c antibody titers in control and BCAP KO mice immunized with 10 µg inactivated H1N1 PR/8 influenza virus per mouse at 7,14,21,28, and 35 days after immunization. (**F-G)** Serum anti-NPCGG IgM or IgG2c antibody titers in control and BCAP KO mice immunized with 50 µg of NP-CGG in combination with Imiquimod-SE (10 µg) per mouse at 7,14,21,28, and 35 days after immunization. All data points represent individual mice with mean. P values of less than 0.05 are shown as *$p < 0.05$; **$p < 0.01$; ***$p<0.001$; calculated with Two-way ANOVA with multiple comparison test

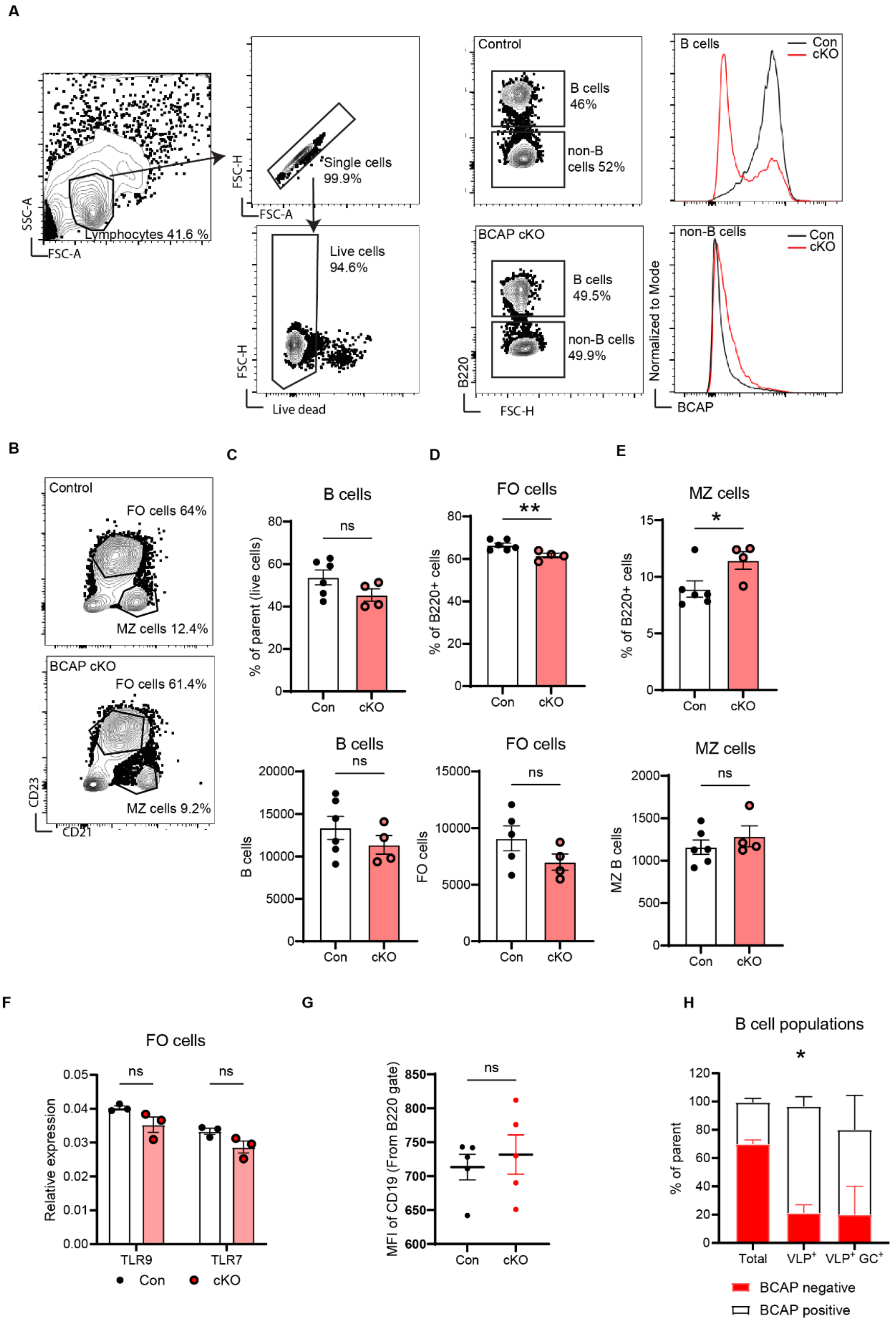

A
SSC-A
FSC-A
Lymphocytes 41.6 %
FSC-H
FSC-A
Single cells 99.9%
Live cells 94.6%
FSC-H
Live dead
Control
B cells 46%
non-B cells 52%
BCAP cKO
B cells 49.5%
non-B cells 49.9%
B220
FSC-H
B cells
Con
cKO
non-B cells
Normalized to Mode
BCAP
B
Control
FO cells 64%
MZ cells 12.4%
BCAP cKO
FO cells 61.4%
MZ cells 9.2%
CD23
CD21
C
B cells
ns
% of parent (live cells)
Con
cKO
B cells
D
FO cells
**
% of B220+ cells
FO cells
E
MZ cells
*
% of B220+ cells
MZ cells
MZ B cells
F
FO cells
Relative expression
TLR9
TLR7
Con
cKO
G
MFI of CD19 (From B220 gate)
H
B cell populations
% of parent
Total
VLP+
VLP+ GC+
BCAP negative
BCAP positive

Fig. S2. B cell subpopulations of BCAP conditional KO.

(A) Gating strategy and analysis of BCAP expression on B and non-B cells in BCAP $^{flox/flox}$ CD19^{cre} mice. Histograms show BCAP expression in B cells or non-B cell gates. **(B)** B cells were gated as B220+ cells and then divided into follicular (FO) and marginal zone (MZ) B cells based on CD23 and CD21 staining. **(C-E)** Percentage (top row) and count (bottom row) of B cells, FO, and MZ cells in BCAP $^{flox/flox}$ CD19^{cre} mice based on the gating strategy in B. All data points represent individual mice (n=6 for control, n=4 for cKO mice per group) with mean and SEM. **(F)** Levels of TLR9 and TLR7 in control and KO Follicular B cells were measured by qPCR. Gene expression was calculated relative to the housekeeping gene *ACTB*. One graph representative of 4 experiments. Dots show technical replicates. (**G**) MFI of CD19 from control and cKO mice after VLP immunization in the B220 gate. Five mice per condition. (H) Antigen-specific cells from the spleen of cKO mice were measured by flow cytometry and classified by their level of BCAP expression, data from 5 different mice. Mann–Whitney U test (C-G), and One-way ANOVA comparing BCAP positive to BCAP negative cells (H) *P < 0.05; **P < 0.01, ***P<0.001, ns= non-significant.

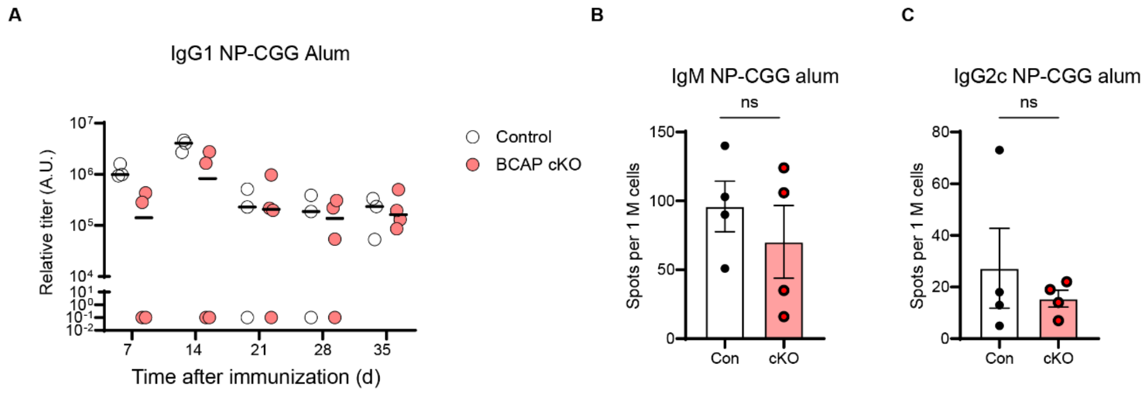

Fig. S3. Levels of antigen-specific antibodies in cKO mice after NP-CGG alum immunization.

(A) Serum anti-NP-CGG IgG1 antibody titers in control and BCAP cKO mice, immunized with 50 µg of NP-CGG in combination with alum (1:1) per mouse at 7,14,21,28, and 35 days after immunization. **(B-C)** Antigen-specific IgM and IgG2c plasma cells enumerated by ELISpot assay on bone marrow cells from control or BCAP cKO mice harvested after immunization. All data points represent individual mice. Graphs show representative data from one of two independent experiments yielding similar findings. Two-way ANOVA with multiple comparisons (A) and Mann-Whitney U (B-C) tests were performed, ns= non-significant.

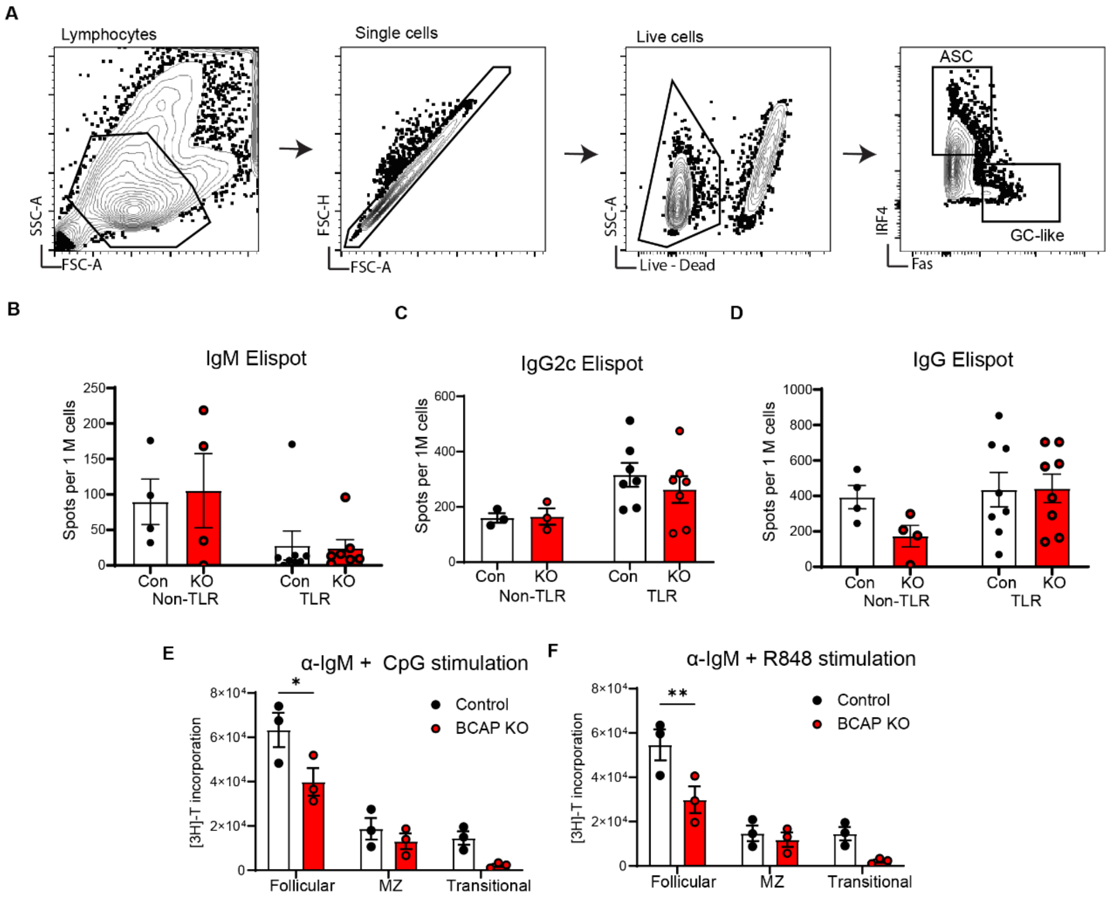

Fig. S4. BCAP KO cell capacity of differentiation and proliferation.

(A) Gating strategy for the plasma cell differentiation assay. (B-D) ELISPOT analysis of antibody-secreting cells after eight days of differentiation culture showing the number of IgM, IgG2c, and IgG spots per 1 million cells. Each point represents one culture from a different mouse. n=4 mice per group in non-TLR, n=8 mice in TLR conditions. **(E-F)** Sorted spleen Follicular ($B220^+CD24^+CD23^{hi}CD21^+$), Marginal Zone ($B220^+CD24^+CD23^{low}CD21^{hi}$) Transitional B cells ($B220^+CD21^{low}CD24^{hi}$) from BCAP KO and control mice after TLR ligands (CpG 2μM, R848 5 μg/ml) and α-IgM (10 μg/ml) stimulation. Proliferation was measured by [3H]-thymidine incorporation and is expressed as mean ± SEM for 3 independent experiments combined. P values of less than 0.05 are shown. *P < 0.05; **P < 0.01, ***P<0.001 by two-way ANOVA with multiple comparison test

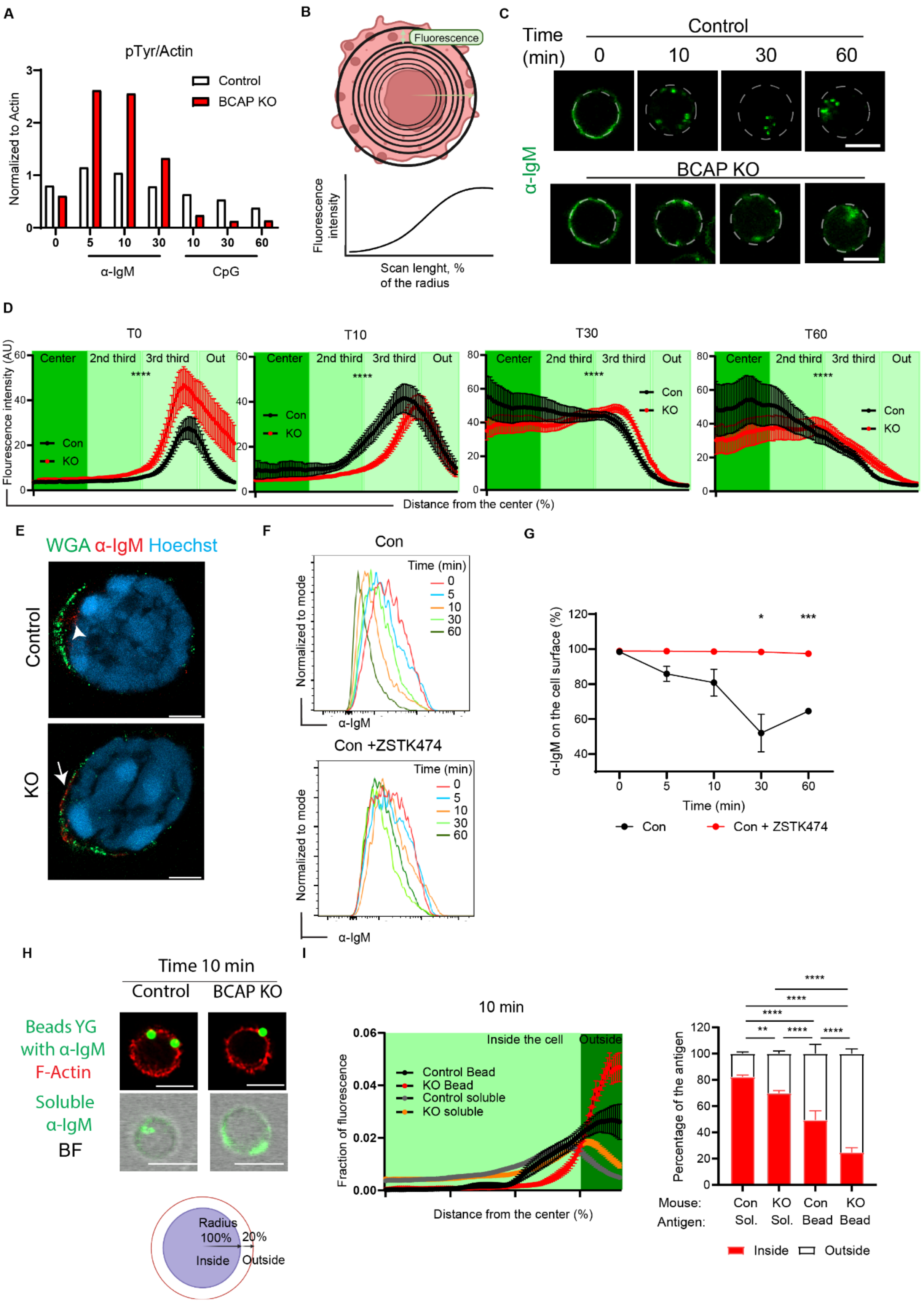
A
pTyr/Actin
Normalized to Actin
Control
BCAP KO
α-IgM
CpG
B
Fluorescence
Fluorescence intensity
Scan lenght, % of the radius
C
Time (min)
Control
0
10
30
60
α-IgM
BCAP KO
D
T0
T10
T30
T60
Center
2nd third
3rd third
Out
Con
KO
Flourescence intensity (AU)
Distance from the center (%)
E
WGA α-IgM Hoechst
Control
KO
F
Con
Time (min)
Normalized to mode
α-IgM
Con +ZSTK474
G
α-IgM on the cell surface (%)
Time (min)
Con
Con + ZSTK474
H
Time 10 min
Control
BCAP KO
Beads YG with α-IgM
F-Actin
Soluble α-IgM
BF
Radius 100%
20%
Inside
Outside
I
10 min
Inside the cell
Outside
Control Bead
KO Bead
Control soluble
KO soluble
Fraction of fluorescence
Distance from the center (%)
Percentage of the antigen
Mouse:
Antigen:
Con Sol.
KO Sol.
Con Bead
KO Bead
Inside
Outside

Fig. S5. BCR signal and endocytosis in control, BCAP KO cells and PI3K-inhibited cells.

(**A**) Quantification of the lanes highlighted in red from Western Blot in Figure 4A. **(B)** Scheme of radial intensity quantification using ClockScan in FIJI. **(C)** Representative confocal images of BCAP KO or control splenic B cells labeled with 10 mg/ml of α-IgM-Biotin for the indicated time points. Cells were fixed and stained with streptavidin-AF88 (green). Images are shown as a single plane. Dotted lines show the contour of the cell based on the Bright Field. Scale bar = 5 µm. N = 2. **(D)** Radial quantification of normalized α-IgM fluorescence from BCAP KO and control B cells relative to inner cell radius 81-84 cells per condition. Data from 1 of 2 different experiments yielding similar results is shown. Kolmogorov–Smirnov test. **** $p < 0.0001$. **(E)** Expansion microscopy images of Control and KO cells after 10 min of α-IgM incubation showing one plane of splenic B cells labeled with α-IgM (red), WGA (green), or Hoechst (blue). Representative image from 1 of 2 different experiments yielding similar results. Scale bar: 10 µm. **(F-G)** Histogram and graphs, from control cells with or without 30 min pre-treatment with PI3K inhibitor ZSTK-474 (1 µM), showing the percentage of surface α-IgM$^+$ cells at different time points after IgM stimulation. Data from 1 of 2 different experiments yielding similar results is shown. Two-way ANOVA with multiple comparison test. (H) Up: Confocal microscopy of control and KO splenic B cells stained with phalloidin (red) with a fluorescent bead (green) conjugated with α-IgM and cells with a soluble α-IgM (green) and their Bright Field (grayscale) after 10 min of incubation with their respective ligand. Images are shown as a single plane. Down: scheme of areas used in the quantification of the following figures (I) Quantification of the images in H. Left measurement of the fraction of the total fluorescence of α-IgM conjugated bead or soluble (sol.) α-IgM as a function of distance from the center. Right: the percentage from the total fluorescence inside or outside of the cell. Data combined from 2 different experiments (cells per condition: Control Bead = 20, Control KO = 27, Soluble control = 57, Soluble KO = 48).

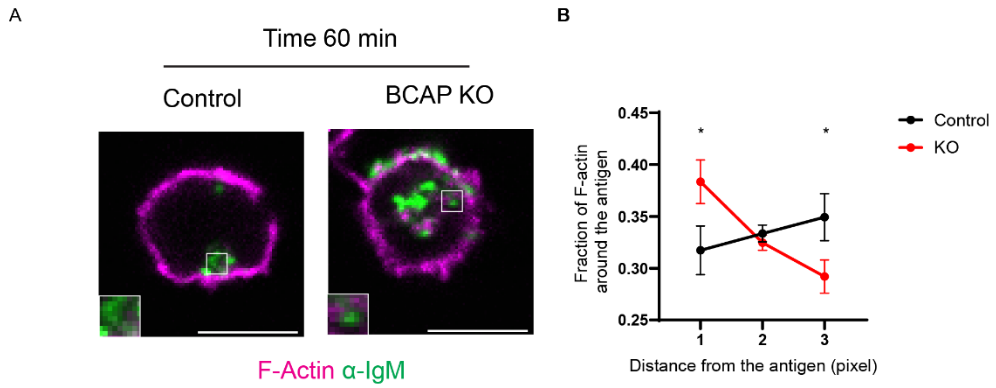

Fig. S6. Role of BCAP recruiting ARP2/3 to the antigen-cell interface

(A) Confocal images from the control and BCAP KO B cells showing actin and α-IgM. Insets highlight soluble α-IgM with and without actin around. (B) Quantification of actin around the antigen in 1 um radius. Representative graph of 2 different experiments yielding similar conclusions. 7 cells per condition. One-way ANOVA, *$P < 0.05$

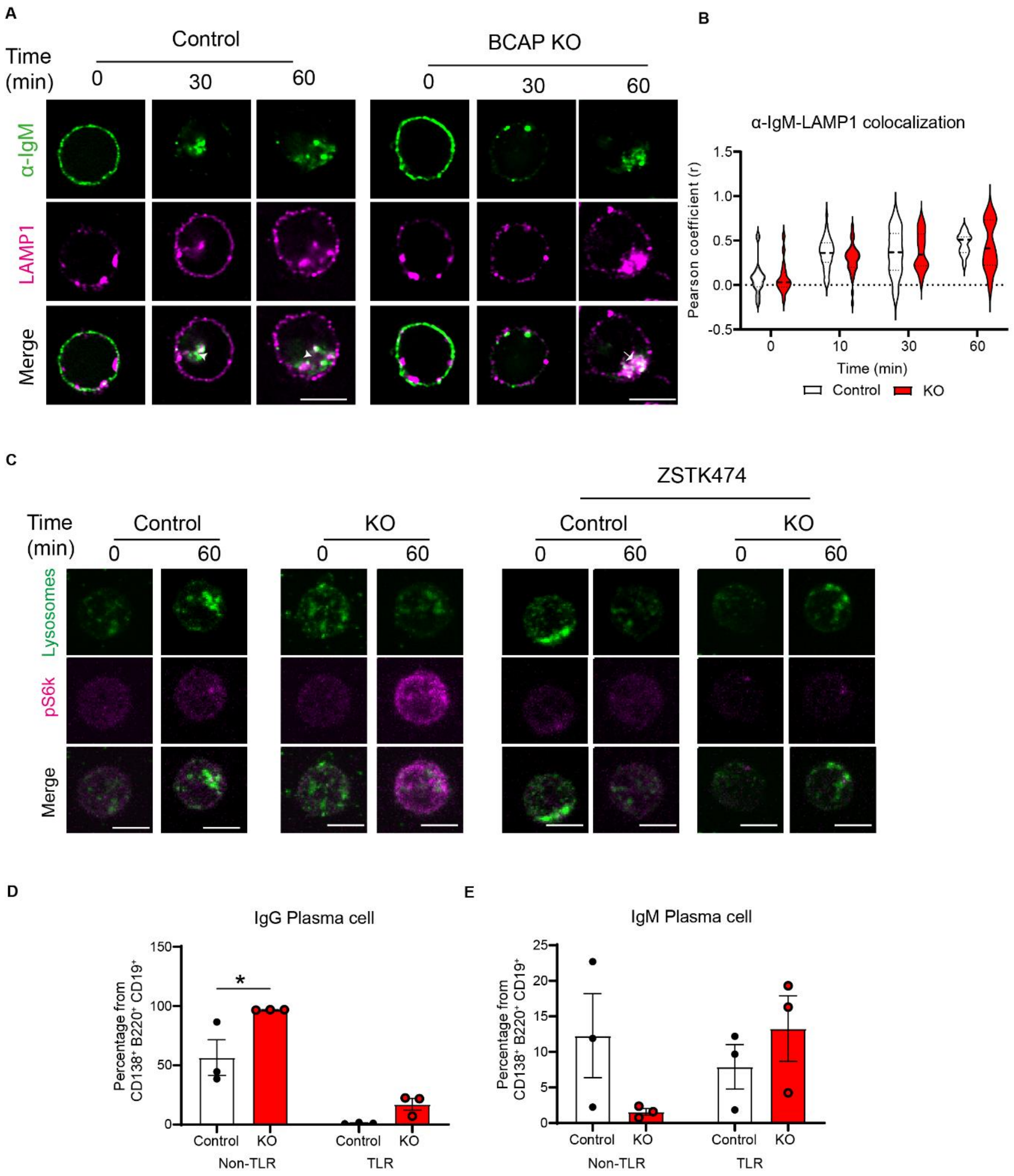

Fig. S7. Effects of lysosomal positioning in mTOR signaling and plasma cell differentiation.

(A) Representative confocal images of BCAP KO or control splenic B cells resting or activated with 10 mg/ml of α-IgM-Biotin for the indicated time points. Cells were fixed and stained with streptavidin-AF488 (green) and LAMP1 (magenta). Images are shown as a single plane. Scale bar = 5 μm. (B) Pearson coefficient between α-IgM and LAMP1.

Graph and images from 1 of 2 independent experiments yielding similar conclusions. 111-102 cells per group **(C)** Confocal Z-project of max intensity in primary B cells from control and KO mice treated 30 min before with PI3K inhibitor ZSTK474 1 μM. Scale bar = 5 μm. Data from 1 of 2 different experiments yielding similar results is shown. **(D-E)** Bar graph showing the percentage of intracellular IgG^+ or IgM^+ cells within the $CD19^+CD138^+$ gate for analysis of isotype-specific plasma cells at eight days post activation. Every point represents 1 biological replicate. (n=3 mice per group in non-TLR, n=3 mice in TLR conditions). Two-way ANOVA with Sidak's multiple comparison test. * $P < 0.05$.
